# Patterns and Drivers of Household Sanitation Access and Sustainability in Kwale County, Kenya

**DOI:** 10.1021/acs.est.0c05647

**Published:** 2021-04-07

**Authors:** Hugo Legge, Katherine E. Halliday, Stella Kepha, Carlos Mcharo, Stefan S. Witek-McManus, Hajara El-Busaidy, Redempta Muendo, Th’uva Safari, Charles S. Mwandawiro, Sultani H. Matendechero, Rachel L. Pullan, William E. Oswald

**Affiliations:** † Faculty of Infectious and Tropical Diseases, London School of Hygiene & Tropical Medicine, London WC1E 7HT, United Kingdom; ‡ Eastern and Southern Africa Centre of International Parasite Control, Kenya Medical Research Institute, P.O. Box 54840-00200, Nairobi, Kenya; § Department of Health, County Government of Kwale, P.O. Box 4-80403, Kwale, Kenya; ∥ Division of Vector Borne and Neglected Tropical Diseases Unit, Ministry of Health, P.O. Box 30016-00100, Nairobi, Kenya

## Abstract

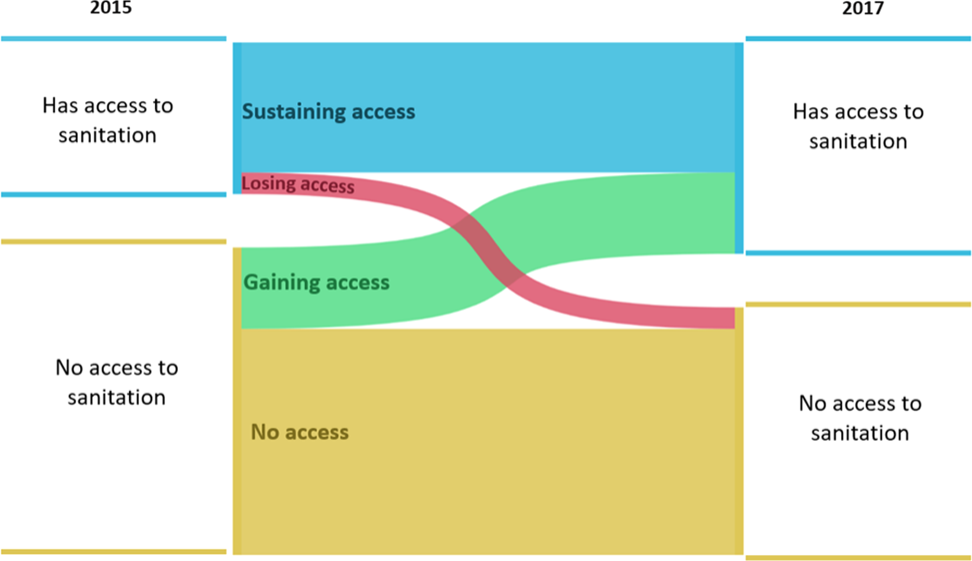

Many sanitation interventions suffer from poor sustainability. Failure to maintain or replace toilet facilities risks exposing communities to environmental pathogens, yet little is known about the factors that drive sustained access beyond project life spans. Using data from a cohort of 1666 households in Kwale County, Kenya, we investigated the factors associated with changes in sanitation access between 2015 and 2017. Sanitation access is defined as access to an improved or unimproved facility within the household compound that is functional and in use. A range of contextual, psychosocial, and technological covariates were included in logistic regression models to estimate their associations with (1) the odds of sustaining sanitation access and (2) the odds of gaining sanitation access. Over two years, 28.3% households sustained sanitation access, 4.7% lost access, 17.7% gained access, and 49.2% remained without access. Factors associated with increased odds of households sustaining sanitation access included not sharing the facility and presence of a solid washable slab. Factors associated with increased odds of households gaining sanitation access included a head with at least secondary school education, level of coarse soil fragments, and higher local sanitation coverage. Results from this study can be used by sanitation programs to improve the rates of initial and sustained adoption of sanitation.

## Background

Sustainable Development Goal 6 challenges the global community to achieve universal, sustainable, and equitable access to safe water, sanitation, and hygiene (WASH) by 2030.^1^ Despite this target, 2.3 billion people still lack access to basic sanitation and 892 million continue to practice open defecation.^2^ Although progress has been made in improving worldwide access to sanitation facilities over the past 2 decades, this progress has been slow and gains have not been evenly distributed among the left-behind countries.^3^ Of the 123 countries that currently have less than 95% access to basic sanitation, only 14 are on track to achieve universal coverage by 2030.^2^ Securing long-term access to sanitation in left-behind communities not only requires improving the rates of initial adoption of sanitation but also ensuring that gains made in sanitation access are sustained over an extended time period or, ideally, indefinitely.^4^ When adoption rates are low, or access cannot be sustained, communities may resort to use of unhygienic and unsafe sanitation facilities or open defecation. Poor uptake and sustainability not only impedes progress toward universal access, exposing or re-exposing communities to fecal pathogens deposited in the environment, but it also represents a hugely inefficient use of resources in a sector that is facing considerable funding shortages.^5^

The contextual and psychosocial settings in which sanitation interventions are delivered (for example, the socioeconomic status of a community or the existence of cultural taboos surrounding disposal of feces) have been demonstrated to play a significant role in influencing the levels of initial and sustained adoption of sanitation.^6^ Many studies have previously investigated the factors that prohibit or promote initial adoption of sanitation^7−16^ and have shown that community demand for sanitation services, often predicated on concerns over privacy, safety, social standing, and health, is a crucial foundation for high levels of adoption.^15,16^ Conversely, economic constraints, lack of household tenure, and the prohibitive cost of latrine construction are widely referenced as common barriers to adoption^7−11^ as is the limited availability of suitable ground to construct latrines due to high population density^10^ or adverse environmental conditions.^17^ In the context of sanitation interventions, programmatic characteristics, such as type of intervention and duration of follow-up, have also been previously shown to be an important factor influencing the initial adoption of sanitation.^18^ At the governmental level, systems analyses and process evaluations have demonstrated that strong institutional support for sanitation programs, replete with sufficient funding at the local and national levels, is crucial for the success of sanitation interventions.^19,20^

While the evidence base covering the local contextual, psychosocial, and technological factors that influence sustained adoption of sanitation is more sparse,^15^ some previous studies have examined sustained adoption as an outcome (i.e., continued household access to sanitation over a given time period). Low quality or poorly contextualized sanitation infrastructure, cultural barriers prohibiting the emptying of latrines, and limited access to the materials and expertise required to maintain facilities have all been found to be associated with poor levels of sustained access.^10,18,21−27^ Weakening demand over time for sanitation services and failure to properly embed behavior change messaging in communities have also been cited as further barriers to achieving sustained adoption.^23^ With the exception of papers by Crocker et al.^21^ and Orgill-Meyer et al.,^22^ these previous studies are mostly drawn from gray literature,^18,23,24^ are cross-sectional, and do not follow household sanitation access longitudinally,^25−27^ or are qualitative in methodology.^10,25^ Therefore, there is a need for further quantitative evidence from longitudinal studies on the contextual, psychosocial, and technological factors that are associated with sustained adoption of sanitation.

In this study we examine the local drivers of change in sanitation access among a cohort of households in Kwale County, Kenya, who were enrolled in the TUMIKIA trial between 2015 and 2017.^28−30^ By following household sanitation access longitudinally, this study contributes to the evidence base on factors that are associated with both initial adoption and sustained adoption of sanitation in southeastern Kenya.

## Methods

### Study Area and Population

This study uses data from a retrospectively compiled cohort of 1666 households enrolled in the TUMIKIA trial that took place between 2015 and 2017 in Kwale County, located in southern coastal Kenya. The county has a population of approximately 866 820, 80% of whom belong to the Mijikenda ethnic group, with other ethnic groups including Digo and Duruma.^31^ The majority of the population (75%) are located in rural communities and are primarily dependent on subsistence farming of maize and cassava.^32,33^ With an estimated 47% of the population living below the poverty line, Kwale has a higher poverty rate than the national average in Kenya.^34^ The climate and geography are heterogeneous across the county and include a low-lying coastal area, an elevated belt running north to south through the middle of the county, and a drier highland area in the west. Although the number of households with access to improved sanitation in Kwale increased from 37 to 57% between 2014 and 2019, open defecation decreased by only 9% over the same period and remains high with 32% reporting no access to any kind of sanitation facility.^33,35,36^ According to the Ministry of Health records, between June 2014 and February 2017, community-led total sanitation (CLTS) “triggerings” (meetings, usually conducted at the village level, where the community’s interest in ending open defecation is stimulated) were facilitated in 62 communities in Kwale County as part of the Kenyan Government’s National Open Defecation Free campaign.^37,38^

### Study Design

Details of the TUMIKIA trial have been previously described.^28,29^ In brief, TUMIKIA was a cluster-randomized, controlled trial evaluating the effectiveness of three alternate mass treatment strategies for controlling soil-transmitted helminths (annual school-based deworming, annual community-wide deworming, and biannual community-wide deworming). The evaluation consisted of repeat cross-sectional surveys conducted with 225 households randomly selected in each of the 120 clusters (broadly equivalent to the “community-units” administrative unit) across the three study arms. This analysis comprises a retrospectively compiled cohort of households within the biannual treatment arm (40 clusters) that were randomly selected and surveyed at both the 2015 and 2017 cross-sectional surveys, with all households that had records from both surveys included in this study’s cohort. Matching household IDs with GPS coordinates more than 100 m apart between 2015 and 2017 surveys were excluded from the analysis as they were assumed to have moved residences during the study period or be the result of an incorrect match (Figure S1).

### Ethical Approval

Written informed consent was obtained from adult representatives of participating households. Where no literate household member was available, the consent sheet was read to the respondent in the presence of an impartial literate witness. Following this, the respondent provided a thumbprint, which was countersigned by the witness. Written informed consent was also sought from adults (≥18 years) selected to complete the individual-level questionnaire. Parental consent was sought for individuals aged 2 to 17 years, and written assent was additionally obtained from children aged 13 to 17 years. All information and consent procedures were conducted in Kiswahili. The TUMIKIA trial protocol was approved by the Kenya Medical Research Institute and National Ethics Review Committee (SSC No. 2826) and the London School of Hygiene & Tropical Medicine (LSHTM) Ethics Committee (7177). This secondary analysis was approved by the LSHTM ethics committee (22504).

### Data Collection

The first survey took place from March to May 2015, and the second survey took place from March to May 2017. Household-level wealth measures and WASH indicators were collected using standard questionnaires. Observations of sanitation facilities located within the compound were conducted using standard checklists.^39^ All data were collected and global positioning system coordinates were recorded at each household using electronic forms via SurveyCTO (Dobility, Inc., Cambridge, MA) on Android smartphones (Google, Mountain View, CA).^39^

### Initial and Sustained Adoption

Households were classed to have sanitation access if the respondent reported the presence of a functioning and currently in-use sanitation facility and the enumerator was able to confirm its presence through direct observation. “Functioning and in-use” was a self-reported measure that included confirmation by the respondent of the facility’s current functionality. Households where enumerators were not able to validate the presence of the facility on the compound (defined as an area where up to 10 households are clustered together) or respondents reported access to a facility located outside of the compound were classed as not having access as this implied nonownership or nonexistence of the facility. Sanitation facilities counted as access included the following: pit latrines without solid washable platforms; pit latrines with solid washable platforms; ventilated improved pit latrines (VIPs); and pour/flush toilets. Unimproved facilities (i.e., pits without a platform) were counted as access to retain latrine quality covariates during analysis and allow results to be generalizable to both improved and unimproved facilities.

To examine both initial and sustained adoption of sanitation between 2015 and 2017, we categorized households on their baseline sanitation access. Within each baseline sanitation access group, we examined a different outcome, representing distinct processes (i.e., initial or sustained adoption). Among households without sanitation access in 2015, we considered the outcome to be gaining access, contrasted with nonadopting and referred to this as the “initial adoption” model. Among households with sanitation access in 2015, we considered the outcome to be sustaining access, contrasted with losing access and referred to this as the “sustained adoption” model. This conceptualization, distinguishing initial adoption from sustained adoption processes between groups defined by baseline household sanitation access, is based on the hypothesis that these outcomes represent distinct processes and that factors underpinning one may not necessarily be relevant or as relevant to the other. This hypothesis has been previously described in both the health psychology and sanitation literature.^6,40,41^

### Covariates

The integrated behavioral model for water, sanitation, and hygiene (IBM WASH) was used as a reference framework to identify candidate contextual, technological, and psychosocial factors for inclusion in the models for the respective processes.^42^ A review of the existing literature and the authors’ knowledge of the study site were employed to finalize the list of candidate predictors within the available 2015 data (Table 1). Contextual environmental covariates related to soil types included sand, silt, and coarse fragment content of the soil. Additional environmental covariates included depth to bedrock, vegetation levels, slope (percent change in elevation over a given distance), average monthly rainfall, depth to groundwater, and aridity levels. Due to the exploratory nature of the analysis and the lack of prevalidated cutoff points to define “high” and “low” categories, environmental covariates were binned based on the distribution of the data using tertiles and then categorized into binary variables as “low/medium” vs “high.” Data sources for these covariates are described in further detail in the Supporting Information (Text S1).

**Table 1:** Selected Covariates from the 2015 Survey to be Included in the Initial and Sustained Sanitation Adoption Models; Presented in the IBM-WASH Framework

	contextual factors	psychosocial factors	technology factors
structural/environmental	^•^sand-soil content		
	^•^coarse fragment-soil content		
	^•^silt-soil content		
	^•^depth to bedrock		
	^•^aridity		
	^•^vegetation		
	^•^average monthly rainfall		
	^•^depth to groundwater		
	^•^slope		
community	^•^distance of household from main road	^•^cluster-level sanitation coverage	
	^•^household status as urban/peri-urban or rural	^•^previous exposure to CLTS triggering event	
household	^•^socio-economic status		- shared vs exclusive access of facility on own compound
	^•^number of household members		
	^•^education level of head of household		
	^•^sex of head of household		
individual			- facility wall type
			- facility platform type
habitual		° use of shared facility on other compound vs use of no facility	- cleanliness of facility
^•^covariates included in both models			
° covariate included only in initial adoption model			
- covariates included only in sustained adoption model			

Individual and household-level contextual covariates included socioeconomic status (SES) (Text S2); number of household members, categorized into tertiles (1–4 members, 5–6 members, and 7+ members); sex of the head of household; highest level of education achieved by the head of household; the locality in which the household was located, dichotomized as urban/peri-urban versus rural; and remoteness of household from a major road. This latter measure was assessed based on GPS coordinates and road network data and dichotomized as greater or less than 4 km from a major road. Previous studies have indicated that proximity to and relationships with other households that have access to sanitation is associated with adoption of sanitation.^43,44^ To measure this phenomenon, we included cluster-level sanitation coverage, calculated as the proportion of households from the full 2015 sample with sanitation access either on or off of the compound, as a proxy for community-level norms and shared values regarding the adoption of sanitation. For the sustained adoption model, we included technological factors related to the sanitation facility in 2015. The factors included are as follows: facility platform type; the cleanliness of the facility (feces visible around the edge of the opening); materials used to construct the walls of the facility’s superstructure; materials used to construct the roof of the facility; and a binary variable indicating whether the household shared the facility with other households or had exclusive access.

For the initial adoption model, no sanitation facility-level covariates were considered due to households only being included if they had no access to sanitation in 2015. However, reporting shared access through the use of a facility located outside of the compound in 2015 was included as this was conceptualized as an indicator demonstrating a habit of latrine use, which could be associated with the odds of gaining sanitation access on the compound over the study period. Village-level exposure to a CLTS triggering event was also included as a covariate in both models to account for programmatic influence on levels of initial and sustained adoption of sanitation.^38^

### Analysis

Of 1666 households included in the study cohort, 1405 households (84.3%) were retained for analysis and 261 (15.6%) were excluded based on discordance between 2015 and 2017 GPS coordinates. Prior to exclusion, sociodemographic and outcome variables were compared between the full study cohort and households with discordant 2015 and 2017 GPS coordinates and were found to have good concordance (Table S1). Variables of interest were tabulated for comparison with values from the full 2015 baseline cross-sectional dataset to examine the cohort’s representativeness. Variables of interest were then tabulated at both survey time points in the cohort dataset to quantify the patterns of change over the course of the study period. To estimate univariate associations between candidate contextual, psychosocial, and technological factors and the outcomes of interest, we used fixed-effects logistic regression models outputting odds ratios (ORs) and 95% confidence intervals (95CIs). Following this, multivariable associations were estimated using multilevel logistic regression models outputting ORs and 95CIs, with random intercepts to account for nesting of households within clusters. Model building for the multivariable analysis followed a predictive, risk-factor analysis approach with the aim of identifying covariates that were significantly associated with the respective outcomes.^45^ Starting with a full model containing all candidate covariates, we selected our final model using a stepwise backward selection process comprising iterative backward elimination followed by forward selection, using Wald tests to generate global *p*-values and with a significance criteria of 0.05.^46,47^ Multicollinearity was assessed in initial models by generating correlation matrices and assessing the correlation coefficients between covariates. All correlation coefficients between variables were found to be less than <0.6, indicating little evidence of strong collinearity between the covariates.^48^

## Results

### Study Population

Among the retained cohort of 1405 households, technological, psychosocial, and sociodemographic factors were broadly equivalent with those of households included in the TUMIKIA 2015 baseline cross-sectional survey (*n* = 23 414). There was some heterogeneity in levels of clusterwide sanitation coverage between cohort and cross-sectional households, and there was a small but significant difference in household-level sanitation access between groups. Additionally, there were some differences between groups among environmental covariates (Table S2). In 2015 in the retained cohort, the majority of households were located in rural localities (75.8%) and located less than 4 km from a main road (75.8%). Mean household size was 5.3 and 65.1% of households had a head who had at least primary level education.

### Patterns of Household Sanitation Access between 2015 and 2017

**Figure 1:**
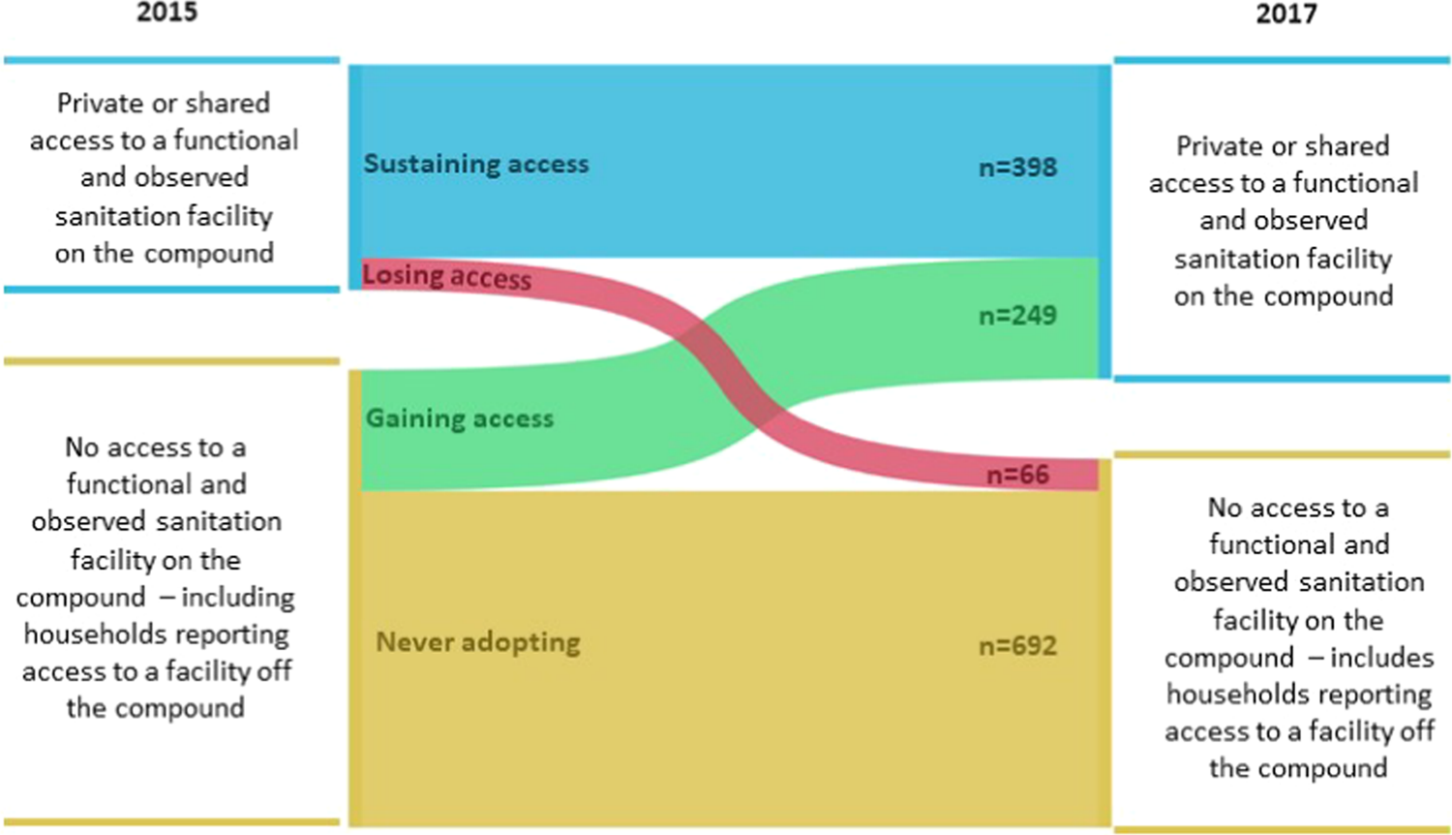
Patterns of household sanitation access among 1405 households in Kwale County, Kenya, between 2015 and 2017.

**Figure 2:**
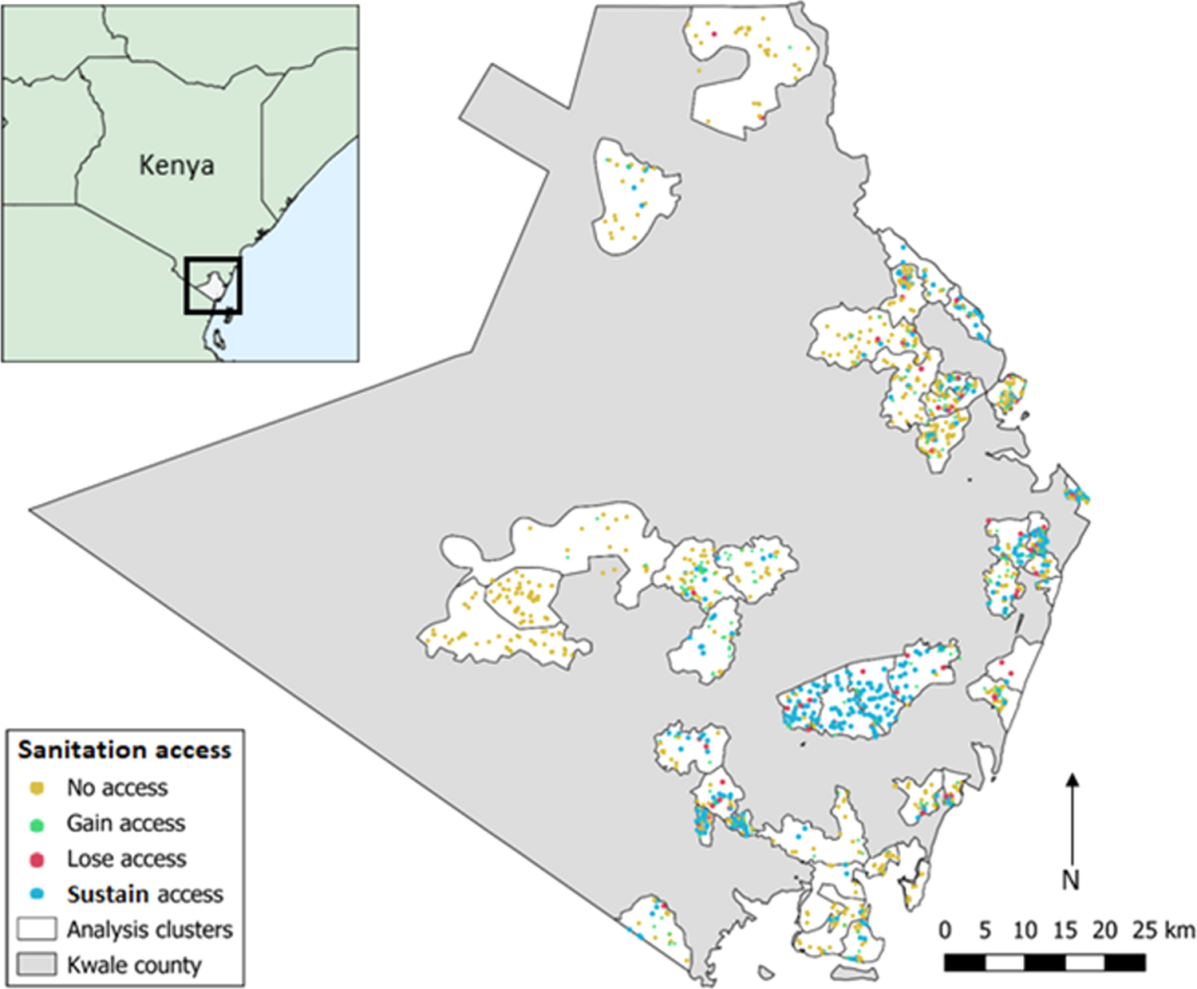
Locations of study households in Kwale County, Kenya, and sanitation access between 2015 and 2017.

Of 1405 included households, 464 (32%) had access to sanitation in 2015, which increased to 647 (46%) by 2017. Overall, between 2015 and 2017, 398 households (28.3%) sustained access to sanitation facilities, 249 (17.7%) gained access, 66 (4.7%) lost access, and 692 (49.3%) did not gain access (Figure 1 and Figure 2). In total, 315 (22.4%) households changed the sanitation status over the study period, resulting in a net increase of 183 households gaining access to a sanitation facility within the study cohort (Table S3).

Despite the increase in overall sanitation access between 2015 and 2017, the proportion of households with access to sanitation reporting exclusive use of the facility fell from 70.4 to 62.8% between 2015 and 2017, and the proportion of sanitation facilities with a solid, washable platform fell slightly from 51.9 to 47.4%. CLTS triggering occurred in 14 study villages, reaching 5.8% of households in the study cohort (Table S2).

With regard to other WASH indicators, self-reported access to an improved water source and access to a handwashing facility within the compound increased from 49.5 to 54% and 5.7 to 16.3%, respectively. Other household, socioeconomic and environmental indicators remained similar over the course of the study period (Table S2).

### Initial Adoption of Sanitation

**Figure 3:**
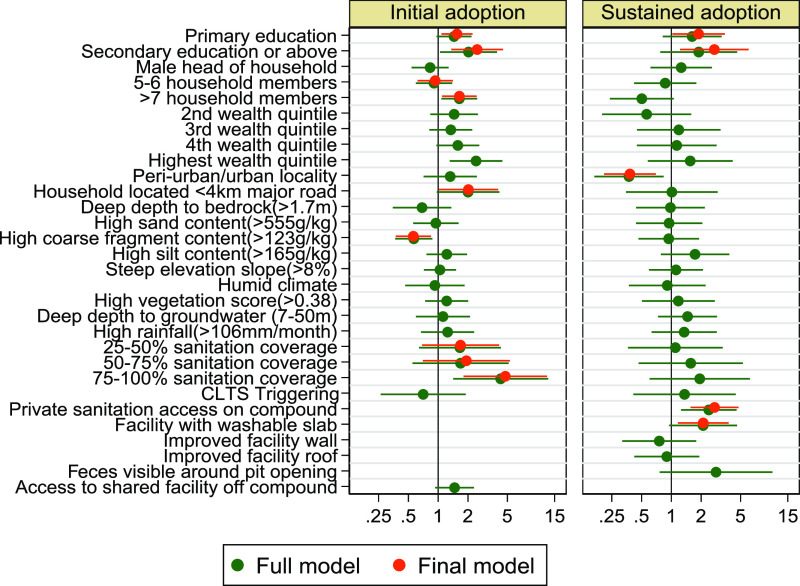
Forest plot with odds ratios and 95% confidence intervals for initial and sustained adoption outcomes from full (green) and final (red) models.

Covariates significantly associated with the initial adoption of sanitation and retained in the final multivariable model included number of household members, education level of the head of household, proximity to a main road, 2015 cluster-level sanitation coverage, and coarse fragment soil content (Table 2 and Figure 3). Higher community sanitation coverage was strongly associated with odds of gaining access to sanitation, comparing households in communities in the highest quartile of coverage to households in communities in the lowest (odds ratio [OR]: 4.77, 95% confidence interval [95CI]: 1.81–12.61). Households where the head had at least secondary school education had 2.48 times the odds of gaining access to sanitation between 2015 and 2017 when compared with households where the head had no education (95CI 1.35–4.52). Households with 7 or more members had 1.64 times the odds of gaining sanitation access than those with 1–4 members (95CI 1.09–2.45), but no difference in odds was observed in households with only 4 or 5 members. Households in areas with high levels of coarse fragments in the soil had lower odds of gaining access than those in areas with medium or low levels (OR 0.56; 95CI 0.37–0.85). Households less than 4 km from a main road had 2.02 times the odds to gain access than those over a 4 km distance (95CI 1.01–4.04).

**Table 2:** Crude and Multivariable Associations between Households Gaining Access (Initial Adoption) to Sanitation over the Study Period and Contextual, Psychosocial, and Technological Factors in 2015

variable	proportion in sample, *n* (%)	proportion with outcome, *n* (%)	crude odds ratio (95 CI)^a^	final model odds ratio (95 CI)^b^	final model *p*-value^c^
SES quintile					
lowest wealth	352 (37.4)	78 (22.2)	1		
2	129 (13.7)	35 (27.1)	1.31 (0.82–2.08)		
3	183 (19.4)	49 (26.8)	1.28 (0.85–1.94)		
4	181 (19.2)	51 (28.2)	1.38 (0.91–2.08)		
highest wealth	96 (10.2)	36 (37.5)	2.11 (1.3–3.42)		
sex of the head of household					
female	228 (24.4)	66 (28.9)	1		
male	706 (75.6)	182 (25.8)	0.86 (0.62–1.2)		
education of the head of household					
no education	370 (39.8)	79 (21.4)	1	1	0.005
primary	484 (52)	136 (28.1)	1.44 (1.05–1.98)	1.55 (1.08–2.23)	
secondary or above	76 (8.2)	32 (42.1)	2.68 (1.59–4.5)	2.48 (1.35–4.52)	
number of household members					
1 to 4	377 (40.1)	88 (23.3)	1	1	0.015
5 to 6	285 (30.3)	69 (24.2)	1.05 (0.73–1.51)	0.93 (0.62–1.41)	
7+	279 (29.6)	92 (33)	1.62 (1.14–2.28)	1.64 (1.09–2.45)	
proximity to main road					
>4 km majroad	235 (25)	23 (9.8)	1	1	0.047
<4 km majroad	706 (75)	226 (32)	4.34 (2.74–6.86)	2.02 (1.01–4.04)	
locality type					
rural	732 (77.8)	174 (23.8)	1		
peri-urban/urban	209 (22.2)	75 (35.9)	1.79 (1.29–2.5)		
aridity index					
semi-arid/sub-humid	444 (47.2)	87 (19.6)	1		
humid	497 (52.8)	162 (32.6)	1.98 (1.47–2.68)		
average monthly rainfall					
low/medium (<106 mm/month)	734 (78)	184 (25.1)	1		
high (>106 mm/month)	207 (22)	65 (31.4)	1.25 (0.67–2.31)		
sand-soil content (2 m)					
low/medium (<555 g/1 kg)	699 (74.3)	163 (23.3)	1		
high (>555 g/kg)	242 (25.7)	86 (35.5)	1.81 (1.32–2.49)		
coarse fragment content (2 m)					
low/medium (<123 cm^3^/dm^3^)	570 (60.6)	178 (31.2)	1		0.006
high (>123 cm^3^/dm^3^)	371 (39.4)	71 (19.1)	0.52 (0.38–0.71)	0.56 (0.37–0.85)	
silt-soil content (2 m)					
low/medium (<165 g/1 kg)	465 (49.4)	151 (32.5)	1		
high (>165g/1 kg)	476 (50.6)	98 (20.6)	0.54 (0.4–0.72)		
depth to bedrock (1.75 m)					
low/medium (<1.7 m)	705 (74.9)	187 (26.5)	1		
high (>1.7 m)	236 (25.1)	62 (26.3)	0.99 (0.71–1.38)		
depth to water table					
0–7 m	302 (32.1)	74 (24.5)	1		
7–50 m	639 (67.9)	175 (27.4)	1.16 (0.85–1.59)		
enhanced vegetation index					
low/medium (<0.38)	691 (73.4)	156 (22.6)	1		
high (>0.38)	250 (26.6)	93 (37.2)	2.03 (1.49–2.78)		
slope (incline)					
low/medium (<8%)	612 (65.6)	156 (25.5)	1		
high (>8%)	321 (34.4)	92 (28.7)	1.17 (0.87–1.59)		
cluster-level sanitation coverage (%)					
0–25	422 (44.8)	66 (15.6)	1	1	0.017
25–50	262 (27.8)	75 (28.6)	2.16 (1.49–3.15)	1.68 (0.69–4.12)	
50–75	118 (12.5)	38 (32.2)	2.56 (1.61–4.09)	1.93 (0.67–5.31)	
75–100	139 (14.8)	70 (50.4)	5.47 (3.58–8.36)	4.77 (1.81–12.61)	
CLTS triggering					
no triggering	890 (94.6)	240 (27)	1		
triggered	51 (5.4)	9 (17.6)	0.58 (0.28–1.21)		
access to shared sanitation on other compound					
no access	786 (83.5)	185 (23.5)	1		
access	155 (16.5)	64 (41.3)	2.28 (1.59–3.27)		

a Odds ratios and 95% confidence intervals were obtained from univariate logistic regression.

b Odds ratios and 95% confidence intervals were obtained from the final adjusted model.

c *p*-Values were derived fromWald tests based on the final adjusted model.

### Sustained Adoption of Sanitation

The final sustained access model included education level of the head of household, urban/rural locality, presence of a solid washable slab, and exclusive/shared access to the sanitation facility (Table 3 and Figure 3). Households with exclusive access to a facility had 2.73 times the odds of sustained access over the study period compared with households sharing their facility with other households (95CI 1.56–4.77). Households owning facilities with a solid, washable slab had 2.10 times the odds of sustained access compared to those without a solid, washable slab (95CI 1.16–3.79). In contrast to households in rural areas, households located in urban or peri-urban localities had 0.38 times the odds of sustained access (95CI 0.21–0.7). Similar to gained access, heads of household who attended at least primary school or at least secondary school both had higher odds of sustaining access than those who had no education (OR 1.88, 95CI 1–3.47; OR 2.72, 95CI 1.22–6.04, respectively).

**Table 3:** Crude and Multivariable Associations between Households Sustaining Access to Sanitation over the Study Period and Contextual, Psychosocial, and Technological Factors in 2015

variable	proportion in sample, *n* (%)	proportion with outcome, *n* (%)	crude odds ratio (95 CI)^a^	final model odds ratio (95 CI)^b^	final model *p*-value^c^
SES quintile					
lowest wealth	71 (15.3)	55 (77.5)	1		
2	40 (8.6)	30 (75)	0.87 (0.35–2.16)		
3	62 (13.4)	51 (82.3)	1.35 (0.57–3.18)		
4	105 (22.6)	91 (86.7)	1.89 (0.86–4.17)		
highest wealth	186 (40.1)	171 (91.9)	3.32 (1.54–7.14)		
sex of the head of household					
female	113 (24.5)	93 (82.3)	1		
male	348 (75.5)	302 (86.8)	1.43 (0.81–2.54)		
education of the head of household					
no education	110 (23.9)	85 (77.3)	1	1	0.028
primary	220 (47.7)	190 (86.4)	1.86 (1.03–3.36)	1.88 (1–3.47)	
secondary or above	131 (28.4)	120 (91.6)	3.21 (1.5–6.87)	2.72 (1.22–6.04)	
number of household members					
1 to 4	176 (37.9)	155 (88.1)	1		
5 to 6	155 (33.4)	134 (86.5)	0.87 (0.42–1.79)		
7+	133 (28.7)	109 (82)	0.5 (0.24–1.06)		
proximity to main road					
>4 km majroad	45 (9.7)	39 (86.7)	1		
<4 km majroad	419 (90.3)	359 (85.7)	0.92 (0.37–2.27)		
locality type					
rural	323 (69.6)	286 (88.5)	1	1	0.002
peri-urban/urban	141 (30.4)	112 (79.4)	0.5 (0.29–0.85)	0.38 (0.21–0.7)	
aridity index					
semi-arid/sub-humid	87 (18.8)	72 (82.8)	1		
humid	377 (81.3)	326 (86.5)	1.33 (0.71–2.5)		
average monthly rainfall					
low/medium (<106 mm/month)	294 (63.4)	246 (83.7)	1		
high (>106 mm/month)	170 (36.6)	152 (89.4)	1.65 (0.92–2.94)		
sand-soil content (2 m)					
low/medium (<555 g/1 kg)	241 (51.9)	198 (82.2)	1		
high (>555 g/kg)	223 (48.1)	200 (89.7)	1.89 (1.1–3.25)		
coarse fragment content (2 m)					
low/medium (<123 cm^3^/dm^3^)	328 (70.7)	289 (88.1)	1		
high (>123 cm^3^/dm^3^)	136 (29.3)	109 (80.1)	0.54 (0.32–0.93)		
silt-soil content (2 m)					
low/medium (<165 g/1 kg)	350 (75.4)	300 (85.7)	1		
high (>165g/1 kg)	114 (24.6)	98 (86)	1.02 (0.56–1.87)		
depth to bedrock (1.75 m)					
low/medium (<1.7 m)	283 (61.4)	235 (83)	1		
high (>1.7 m)	178 (38.6)	160 (89.9)	1.82 (1.02–3.24)		
depth to water table					
0–7 m	160 (34.5)	130 (81.3)	1		
7–50 m	304 (65.5)	268 (88.2)	1.72 (1.01-2.91)		
enhanced vegetation index					
low/medium (<0.38)	229 (49.4)	185 (80.8)	1		
high (>0.38)	235 (50.6)	213 (90.6)	2.3 (1.33-3.98)		
slope (incline)					
low/medium (<8%)	281 (61.4)	238 (84.7)	1		
high (>8%)	177 (38.6)	154 (87)	1.21 (0.7-2.09)		
cluster-level sanitation coverage (%)					
0–25	45 (9.7)	36 (80)	1		
25–50	88 (19)	68 (77.3)	0.85 (0.35-2.06)		
50–75	82 (17.7)	68 (82.9)	1.21 (0.48-3.08)		
75–100	249 (53.7)	226 (90.8)	2.46 (1.05-5.73)		
CLTS triggering					
no triggering	433 (93.3)	373 (86.1)	1		
triggered	31 (6.7)	25 (80.6)	0.67 (0.26-1.7)		
exclusive access to facility					
shared access on compound	137 (29.5)	106 (77.4)	1	1	<0.001
exclusive access on compound	327 (70.5)	292 (89.3)	2.44 (1.43-4.15)	2.73 (1.56-4.77)	
facility with durable, washable slab					
without slab	223 (48.1)	184 (82.5)	1	1	0.014
with slab	241 (51.9)	214 (88.8)	1.68 (0.99-2.85)	2.1 (1.16-3.79)	
feces visible around latrine opening					
no feces present	416 (89.7)	353 (84.9)	1		
feces present	48 (10.3)	45 (93.8)	2.68 (0.81-8.88)		
facility wall					
no wall/natural materials	269 (58)	229 (85.1)	1		
improved materials	195 (42)	169 (86.7)	1.14 (0.67-1.93)		
facility roof					
no roof/natural materials	240 (51.7)	202 (84.2)	1		
improved materials	224 (48.3)	196 (87.5)	1.32 (0.78-2.23)		

a Odds ratios and 95% confidence intervals were obtained from univariate logistic regression.

b Odds ratios and 95% confidence intervals were obtained from the final adjusted model.

c *p*-Values were derived from Wald tests based on the final adjusted model.

## Discussion

Our results demonstrate that certain facility characteristics such as use of a slab made from durable materials and exclusive household access are associated with sustained adoption of sanitation. Community-level psychosocial factors, represented in this study by 2015 community-wide sanitation coverage, were found to be associated with initial adoption, indicating that social norms surrounding the adoption of sanitation were an important driver of households gaining sanitation access. A range of contextual factors at the household, community, and environmental levels were also associated with both initial and sustained sanitation adoption. Most notably, households with heads who had at least primary school-level education had higher odds of sustaining and gaining access to sanitation between 2015 and 2017 than those with no education.

### Technological Factors

The lack of an association between the quality of materials used to construct the walls and roof of the superstructure and sustainability of sanitation access suggests that either manufactured materials are no more durable than natural materials in the context of latrine life spans or facility superstructures built with natural materials, though potentially less durable, may be more likely to be re-erected after suffering damage or collapse. Evidence from the CLTS literature supports the latter. Previous studies have found that while superstructures constructed with durable materials are associated with increased facility life spans, accessibility and affordability of materials are key considerations for whether a facility will be built in the first place or replaced after reaching the end of its life span.^23,24^

In contrast to the materials used to construct the facility walls and roof, the presence and type of platform in the facility was associated with increased odds of sustaining access over the study period. Specifically, households with access to facilities with platforms built from durable, manufactured materials had higher odds of sustaining access than households with no platform or a platform built with natural materials. These results suggest that programs should approach latrine quality pragmatically, promoting the use of manufactured materials for the platform, but taking into consideration the availability and cost of such materials when constructing the superstructure, so as to facilitate user-led repair and reconstruction when facilities become damaged or reach the end of their life span. An example of where this has already been trialed can be found in Kilifi, Kenya, where local manufacturing of solid sanitation platforms was incorporated into an urban CLTS project with high levels of recipient acceptability reported.^49^

We found that self-reported exclusive access to a facility was predictive of sustaining access to sanitation over the study period. This result is supported by findings from previous studies that have shown that shared access is associated with both lower user satisfaction and lower likelihood of being used.^50,51^ However, to our knowledge, no previous study has identified this factor as being associated with sustainability of access.

Exclusive access to a facility and the presence of a slab differentiate “unimproved”, “limited”, and “basic” levels of access to sanitation on the Joint Monitoring Program’s (JMP) sanitation service ladder. Our findings that exclusive household access to a facility and the use of a facility with a solid washable slab are associated with increased odds of sustained adoption suggest that in addition to the health, dignity, and convenience of users, these levels should be considered relevant to sustainability of access, with unimproved and limited being slippery rungs from which households can fall down and basic representing a more secure level of access.

More broadly, there is a continued lack of consensus within the WASH sector over how to evaluate the sustainability of household sanitation services in resource-limited settings, with at least six different frameworks in current usage.^52−57^ Our results highlight the importance of including indicators that measure technical components of sanitation facilities such as quality of materials and ease of reconstruction in such frameworks, which not all frameworks currently include.^58^

### Psychosocial Factors

Our results show that levels of community-wide access to sanitation are associated with household-level initial adoption of sanitation. This finding suggests that psychosocial factors such as community norms regarding the adoption of sanitation may play a role in promoting or inhibiting the initial adoption of sanitation. This finding is supported by previous studies in the environmental health literature, which have utilized the concepts of behavior settings and social networks to demonstrate how social norms can influence WASH behaviors through settings or environments that discourage or promote usage.^21,43,44,59−61^ In addition to the psychosocial component, high levels of sanitation access at the community level over a sustained time period may also facilitate growth in the sanitation service chain, creating an economic and technological environment, which can more easily facilitate the construction, maintenance, and emptying of sanitation facilities. Previous studies have linked the absence of such a service chain to poor sustainability of sanitation outcomes.^8,18,21^

### Contextual Factors

Our finding that the education level of the head of household was associated with both initial and sustained adoption of sanitation replicates those of previous studies that identified educational attainment of the head of household as drivers of sanitation outcomes.^50,51,62,63^ Similarly, higher numbers of household members have been previously found to be associated with both latrine ownership and reduced levels of open defecation.^14,50^ In this study there was an observed association between larger households and increased odds of gaining access to sanitation, which may reflect the declining acceptability of open defecation as an option for households as numbers of members increase. We hypothesized that the opposite may be the case for sustained adoption, as larger numbers of users could exert greater pressure on existing sanitation facilities, leading to pit capacity being reached more quickly as well as an increased risk of breakdown in facility functionality due to higher levels of usage. This hypothesis was supported by results in the univariate analysis that demonstrated a negative relationship between household size and sustained adoption. However, the relationship was not significant in multivariable analysis, and the variable was not included in the final model for sustained adoption.

In this study, households located in urban and peri-urban areas had lower odds of sustaining access over the study period when compared to households in rural areas. These results support findings from previous studies that have identified barriers to sustaining sanitation access that are unique to urban settings, such as lack of available space to replace nonfunctioning latrines and the difficulty of emptying existing latrines.^49,64,65^ That locality was significantly associated with sustained adoption but not initial adoption of sanitation could indicate that urban households have sufficient space for only a limited number of latrines. This may be because they do not have access to the sanitation service chain necessary to empty, transport, and safely store feces deposited in the latrine, or the available space to build new latrines once current pits reach the capacity.

Among environmental covariates, the level of coarse fragments in the soil was associated with lower odds of gaining access to sanitation. Soils with higher levels of coarse fragments are typically less cohesive and facilitate percolation of water at a more rapid rate than finer soils, which can make latrine construction more difficult and more easily precipitate the flooding and collapse of existing latrines. Although only limited work has been done investigating the relationship between soil type and sanitation outcomes, this result supports findings from a previous study in Ethiopia, which found that households in areas with coarser soil types were less likely to have access to sanitation.^17^ That coarse fragment levels were only predictive of initial adoption may be due to households choosing not to or being unable to construct latrines on land considered to be unsuitable. These results highlight the need for sanitation program implementers to consider not only soil conditions but also the environmental suitability of the latrine designs they recommend. Alternative designs are available for settings with unstable soil, but the rudimentary designs widely promoted through CLTS interventions may remain inaccessible for households located in areas less suitable to traditional pit latrines.

This study follows a retrospective cohort of households over a limited time frame of two years, which is at the lower end of the spectrum over which to examine sustained access, and would ideally be longer. As a result, the possibility that we are presenting and analyzing data that is reflective of repeat cycles of the gaining and losing of sanitation access cannot be entirely ruled out. Although an indicator for facility cleanliness was included as a covariate, we were not able to provide a measure of the levels of ongoing maintenance and proper usage of sanitation facilities, which potentially could have been an important factor predicting sustained adoption. The classification of sanitation access in this study was conservative, with households having to report not only ownership on their own compound but also current functionality and verification through enumerator observation. Consequently, it is possible that we have underestimated sanitation access in the study site, which could have introduced an element of nondifferential misclassification into the analysis. Cluster-level sanitation access, used here as an indicator for social norms regarding the use of sanitation, covered a geographic area that included in some instances villages with heterogeneous levels of sanitation access. As a result, the cluster-level measure may not represent local conditions for each household, but we would expect this nondifferential misclassification to bias our results toward the null. Future investigations hoping to capture this same phenomenon could record local social networks or use complimentary qualitative methods to identify psychosocial factors. There were small but appreciable differences in household and clusterwide sanitation access between the full 2015 survey and the longitudinal sample, which although not relevant to the internal validity of the study may have impacted the generalizability of the findings.

Findings from this study can be used to inform the ongoing implementation of sanitation interventions in Kenya and in other settings with similar sanitation and socioeconomic profiles. Of particular relevance to programs are the results that highlight the strong relationship between both high-quality toilet slabs and exclusive household access to a facility and sustained adoption of sanitation as these learnings are directly applicable to the intervention design. In addition, our findings also highlight the important association that exists between community-wide sanitation coverage and initial adoption of sanitation by households. The 75% sanitation coverage threshold could be used by programs to identify communities at greater risk of nonadoption. Finally, the study also identifies a number of contextual risk factors for lower levels of initial and sustained adoption, including unsuitable soil conditions and urban environments, which could be used by programs to guide allocation of resources to communities at greater risk of poor sanitation outcomes.

## Supporting Information Available

Sociodemographic, environmental, and WASH variables in included households and households dropped from the analysis due to discordance between 2015 and 2017 household GPS coordinates; demographic, socioeconomic, and environmental characteristics of households in 2015 cross-sectional survey and study cohort; demographic, socioeconomic, and environmental characteristics of study cohort households in 2015 and 2017; full and final model outputs measuring associations between households gaining access to sanitation (initial adoption) over the study period and contextual, psychosocial and technological factors in 2015; full and final model outputs measuring associations between households sustaining access to sanitation (sustained adoption) over the study period and contextual, psychosocial, and technological factors in 2015; factor eigenvalues and variance proportions for urban households; rotated factor loadings and unique variances for urban households; factor eigenvalues and variance proportions for rural households; rotated factor loadings and unique variances for rural households; collinearity matrix for covariates included in the sustained adoption model; collinearity matrix for covariates included in the initial adoption model; details on environmental covariate data sources and variable creation; description of variables included in factor analysis to create socioeconomic score; data flow diagram; and environmental soil covariates (PDF)
