# Supplementary material for: Patterns and Drivers of Household Sanitation Access and Sustainability in Kwale County, Kenya
Source: Environ Sci Technol. 2021 Apr 7;55(9):6052–64. doi: 10.1021/acs.est.0c05647 (PMC8154356; doi:10.1021/acs.est.0c05647)
Supplement: Supplementary file 1 — es0c05647_si_001.pdf [file es0c05647_si_001.pdf]

Patterns and drivers of household sanitation access and sustainability in Kwale county, Kenya

Supporting information

Hugo Legge[1*], Katherine E Halliday[1], Stella Kepha[1,2], Carlos Mcharo[2], Stefan S Witek-McManus[1], Hajara El-Busaidy[3], Redempta Muendo[3], Th'uva Safari[2], Charles S. Mwandawiro[2], Sultani H. Matendechero[4], Rachel L Pullan[1], William E Oswald[1]

[1] Faculty of Infectious and Tropical Diseases, London School of Hygiene & Tropical Medicine, London, United Kingdom

[2] Eastern and Southern Africa Centre of International Parasite Control, Kenya Medical Research Institute, Nairobi, Kenya

[3] Department of Health, County Government of Kwale, Kwale, Kenya

[4] Division of Vector Borne and Neglected Tropical Diseases Unit, Ministry of Health, Nairobi, Kenya

*Corresponding author

Number of pages: 24

Number of figures: 2

Number of tables: 11

Figure S1. Data flow diagram

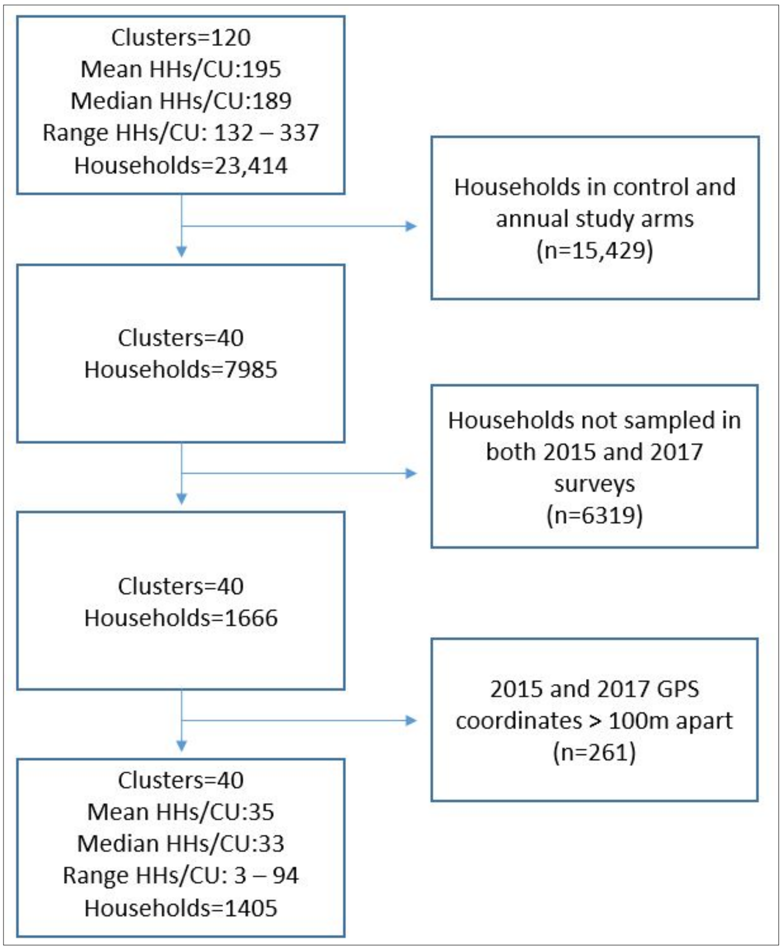

Table S1. Sociodemographic, environmental and WASH variables in the full study sample and in households dropped from the analysis due to discordance between 2015 and 2017 GPS household coordinates.

	2015		2017	
**Variable**	**Full sample, n (%)**	**Dropped households, n (%)**	**Full sample, n (%)**	**Dropped households, n (%)**
**SES quintile**				
Lowest	523 (31.4)	100 (38.3)	454 (27.3)	78 (29.9)
2nd	203 (12.2)	34 (13)	234 (14)	29 (11.1)
3rd	286 (17.2)	41 (15.7)	361 (21.7)	51 (19.5)
4th	327 (19.6)	41 (15.7)	314 (18.8)	62 (23.8)
Highest	327 (19.6)	45 (17.2)	303 (18.2)	41 (15.7)
**Number of household members**				
1 to 4	662 (39.7)	109 (41.8)	468 (28.1)	68 (26.1)
5 to 6	528 (31.7)	88 (33.7)	515 (30.9)	87 (33.3)
7+	476 (28.6)	64 (24.5)	683 (41)	106 (40.6)
**Education of head of households**				
No education	575 (34.8)	95 (36.4)	545 (37)	90 (38.6)
Primary	846 (51.2)	142 (54.4)	717 (48.6)	120 (51.5)
Secondary or above	231 (14)	24 (9.2)	212 (14.4)	23 (9.9)
**Cluster level-sanitation coverage**				
0-25%	577 (34.6)	110 (42.1)	577 (34.6)	110 (42.1)
25-50%	409 (24.5)	59 (22.6)	409 (24.5)	59 (22.6)
50-75%	228 (13.7)	28 (10.7)	228 (13.7)	28 (10.7)
75-100%	452 (27.1)	64 (24.5)	452 (27.1)	64 (24.5)
**Proximity to main road**				
>4km major road	351 (21.1)	71 (27.2)	352 (21.1)	72 (27.6)
<4km major road	1315 (78.9)	190 (72.8)	1314 (78.9)	189 (72.4)
**Locality type**				
Rural	1263 (75.8)	208 (79.7)	1265 (75.9)	212 (81.2)
Peri-urban/urban	403 (24.2)	53 (20.3)	401 (24.1)	49 (18.8)
**Aridity index**				
semi-arid/sub-sumid	644 (38.7)	113 (43.3)	646 (38.8)	115 (44.1)
humid	1022 (61.3)	148 (56.7)	1020 (61.2)	146 (55.9)
**Sand-soil content (2m)**				
low/medium	1139 (68.4)	199 (76.2)	1138 (68.3)	184 (70.5)
high	527 (31.6)	62 (23.8)	528 (31.7)	77 (29.5)
**Coarse fragment content (2m)**				
low/medium	1082 (64.9)	184 (70.5)	1066 (64)	153 (58.6)
high	584 (35.1)	77 (29.5)	600 (36)	108 (41.4)
**Silt-soil content (2m)**				
low/medium	976 (58.6)	161 (61.7)	995 (59.7)	148 (56.7)
high	690 (41.4)	100 (38.3)	671 (40.3)	113 (43.3)
**Distance to bedrock**				
low/medium	1179 (70.9)	191 (73.2)	1169 (70.2)	181 (69.3)
high	484 (29.1)	70 (26.8)	497 (29.8)	80 (30.7)
**Vegetation index**				

low	669 (40.2)	112 (42.9)	672 (40.3)	112 (42.9)
medium	423 (25.4)	60 (23)	440 (26.4)	59 (22.6)
high	574 (34.5)	89 (34.1)	554 (33.3)	90 (34.5)
**Sanitation access**				
No	1128 (67.7)	187 (71.6)	926 (55.6)	168 (64.4)
Yes	538 (32.3)	74 (28.4)	740 (44.4)	93 (35.6)
**Facility with durable, washable slab**				
no	257 (47.8)	34 (45.9)	382 (51.6)	42 (45.2)
yes	281 (52.2)	40 (54.1)	358 (48.4)	51 (54.8)
**Private access to facility**				
shared access on compound	159 (29.6)	22 (29.7)	270 (36.5)	29 (31.2)
private access on compound	379 (70.4)	52 (70.3)	470 (63.5)	64 (68.8)
**Facility roof**				
No roof/natural materials	343 (54.7)	46 (56.1)	419 (50.9)	46 (44.7)
Improved materials	284 (45.3)	36 (43.9)	404 (49.1)	57 (55.3)
**Facility wall**				
No wall/natural materials	369 (58.9)	45 (54.9)	507 (61.6)	60 (58.3)
Improved materials	258 (41.1)	37 (45.1)	316 (38.4)	43 (41.7)

Table S2. Demographic, socioeconomic and environmental characteristics of households in 2015 cross-sectional survey and study cohort

Variable	2015 cross sectional, n (%)	2015 cohort, n (%)	p-value*
**SES quintile**			
Lowest	6617 (30.4)	423 (30.1)	0.962
2nd	2550 (11.7)	169 (12)	
3rd	3932 (18.1)	245 (17.4)	
4th	4382 (20.1)	286 (20.4)	
Highest	4267 (19.6)	282 (20.1)	
**Sex of head of household**			
female	5619 (26.1)	342 (24.5)	0.193
male	15906 (73.9)	1053 (75.5)	
**Number of household members**			
1 to 4	9081 (41.8)	553 (39.4)	0.205
5 to 6	6584 (30.3)	440 (31.3)	
7+	6081 (28)	412 (29.3)	
**Education of head of household**			
No education	7829 (36.5)	480 (34.5)	0.329
Primary	10576 (49.3)	704 (50.6)	
Secondary or above	3060 (14.3)	207 (14.9)	
**Cluster level sanitation coverage**			
0-25%	3972 (18.3)	467 (33.2)	<0.001
25-50%	5291 (24.3)	350 (24.9)	
50-75%	7169 (33)	200 (14.2)	
75-100%	5316 (24.4)	388 (27.6)	
**Proximity to main road**			
>4km major road	3770 (17.3)	280 (19.9)	0.013
<4km major road	17978 (82.7)	1125 (80.1)	
**Locality type**			
Rural	16030 (73.7)	1055 (75.1)	0.254
Peri-urban/urban	5718 (26.3)	350 (24.9)	
**Aridity index**			
semi-arid/sub-sumid	9103 (41.9)	531 (37.8)	0.003
humid	12645 (58.1)	874 (62.2)	
**Average monthly rainfall**			
Low/medium (<106mm/month)	14,624 (67.3)	1,028 (73.1)	<0.001
High (>106mm/month)	7,104 (32.70)	377 (26.83)	
**Sand-soil content (2m)**			
Low/medium (<555g/1kg)	14471 (66.5)	940 (66.9)	0.781
High (>555g/kg)	7276 (33.5)	465 (33.1)	
**Coarse fragment content (2m)**			
Low/medium (<123 cm^3/dm^3)	14544 (66.9)	898 (63.9)	0.022
High (>123 cm^3/dm^3)	7203 (33.1)	507 (36.1)	
**Silt-soil content (2m)**			
Low/medium (<165g/1kg)	14645 (67.3)	815 (58)	<0.001
High (>165g/1kg)	7102 (32.7)	590 (42)	
**Depth to bedrock**			
low/medium (<1.7m)	14467 (67.2)	988 (70.5)	0.011

High (>1.7m)	7070 (32.8)	414 (29.5)	
**Depth to groundwater**			0.02
0-7m	6,506 (29.9)	462 (32.8)	
7-50m	15,222 (70.06)	943 (67.12)	
**Slope**			0.044
Low/medium (<8%)	14,326 (66.8)	893 (64.2)	
High (>8%)	7,112 (33.2)	498 (35.8)	
**Vegetation index**			<0.001
low	6933 (31.9)	557 (39.6)	
medium	7379 (33.9)	363 (25.8)	
high	7436 (34.2)	485 (34.5)	
**CLTS triggering**			0.587
No Triggering	20,553 (94.5)	1,323 (94.6)	
CLTS Triggering	1,195 (5.5)	81 (5.8)	
**Sanitation access**			0.011
No	13826 (63.6)	941 (67)	
Yes	7911 (36.4)	464 (33)	
**Facility with durable, washable slab**			0.682
No	3727 (47.1)	223 (48.1)	
Yes	4189 (52.9)	241 (51.9)	
**Visible faeces around pit opening**			0.899
No faeces present	7915 (90.8)	490 (89.9)	
Faeces present	872 (9.9)	55 (10.1)	
**Private access to facility**			0.423
shared access on compound	2476 (31.3)	137 (29.5)	
private access on compound	5435 (68.7)	327 (70.5)	
**Facility roof**			0.806
No roof/natural materials	4741 (54)	297 (54.5)	
Improved materials	4046 (46)	248 (45.5)	
**Facility walls**			0.030
No wall/natural materials	4805 (54.7)	324 (59.4)	
Improved materials	3982 (45.3)	221 (40.6)	

*P-value outputted from Chi-squared test

Table S3. Demographic, socioeconomic and environmental characteristics of study cohort households in 2015 and 2017

Variable	2015 cohort, n (%)	2017 cohort, n (%)	p-value*
**Education of head of household**			0.418
No education	480 (34.5)	455 (36.7)	
Primary	704 (50.6)	597 (48.1)	
Secondary or above	207 (14.9)	189 (15.2)	
**Number of household members**			<0.001
1 to 4	553 (39.4)	400 (28.5)	
5 to 6	440 (31.3)	428 (30.5)	
7+	412 (29.3)	577 (41.1)	
**Proximity to main road**			>0.999
<4km majroad	1125 (80.1)	1125 (80.1)	
>4km majroad	280 (19.9)	280 (19.9)	
**Sand-soil content (2m)**			0.573
low/medium	940 (66.9)	954 (67.9)	
high	465 (33.1)	451 (32.1)	
**Coarse fragment content (2m)**			0.554
low/medium	898 (63.9)	913 (65)	
high	507 (36.1)	492 (35)	
**Silt-soil content (2m)**			0.219
low/medium	815 (58)	847 (60.3)	
high	590 (42)	558 (39.7)	
**Distance to bedrock**			0.930
low/medium	988 (70.5)	988 (70.3)	
high	414 (29.5)	417 (29.7)	
**Aridity index**			>0.999
semi-arid/sub-sumid	531 (37.8)	531 (37.8)	
humid	874 (62.2)	874 (62.2)	
**Vegetation index**			0.635
low	557 (39.6)	560 (39.9)	
medium	363 (25.8)	381 (27.1)	
high	485 (34.5)	464 (33)	
**Sanitation access**			<0.001
No	941 (67)	758 (54)	
Yes	464 (33)	647 (46)	
**Facility with durable, washable slab**			0.140
no	223 (48.1)	340 (52.6)	
yes	241 (51.9)	307 (47.4)	
**Private access to facility**			0.007
shared access on compound	137 (29.5)	241 (37.2)	
private access on compound	327 (70.5)	406 (62.8)	
**Facility roof**			0.343
No roof/natural materials	297 (54.5)	373 (51.8)	
Improved materials	248 (45.5)	347 (48.2)	
**Facility wall**			0.342
No wall/natural materials	324 (59.4)	447 (62.1)	
Improved materials	221 (40.6)	273 (37.9)	
**Primary water source <30 min from household**			<0.001

No	265 (19)	385 (27.4)	
Yes	1131 (81)	1020 (72.6)	
**Primary water source type**			0.016
Non-improved	709 (50.5)	646 (46)	
Improved	694 (49.5)	759 (54)	
**Access to handwashing station on compound**			<0.001
no	509 (94.3)	603 (83.8)	
yes	31 (5.7)	117 (16.3)	

*P-value outputted from Chi-squared test

Table S4. Full and final model outputs measuring associations between households gaining access to sanitation (initial adoption) over the study period and contextual, psychosocial and technological factors in 2015

Variable	Proportion with outcome, n (%)	Full model odds ratio (95 CI)*	Full model p-value**	Final model odds ratio (95 CI)***	Final model p-value****
**SES quintile**			0.119		
Lowest wealth	78 (22.2)	1.45 (0.83-2.52)			
2	35 (27.1)	1.34 (0.81-2.21)			
3	49 (26.8)	1.58 (0.96-2.6)			
4	51 (28.2)	2.41 (1.31-4.45)			
Highest wealth	36 (37.5)	1.45 (0.83-2.52)			
**Head of household sex**			0.140		
Female	66 (28.9)	1			
Male	182 (25.8)	0.83 (0.54-1.28)			
**Education of head of household**			0.003		0.005
No education	79 (21.4)	1		1	
Primary	136 (28.1)	1.45 (0.96-2.17)		1.55 (1.08-2.23)	
Secondary or above	32 (42.1)	2.03 (1.04-3.94)		2.48 (1.35-4.52)	
**Number of household members**			0.012		0.015
1 to 4	88 (23.3)	1		1	
5 to 6	69 (24.2)	0.91 (0.59-1.39)		0.93 (0.62-1.41)	
7+	92 (33)	1.64 (1.08-2.48)		1.64 (1.09-2.45)	
**Proximity to main road**			0.046		0.047
>4km majroad	23 (9.8)	1		1	
<4km majroad	226 (32)	2 (0.97-4.16)		2.02 (1.01-4.04)	
**Locality type**			0.300		
Rural	174 (23.8)	1			
Peri-urban/urban	75 (35.9)	1.32 (0.71-2.46)			
**Aridity index**			0.570		
semi-arid/sub-sumid	87 (19.6)	1			
Humid	162 (32.6)	0.93 (0.46-1.85)			
**Average monthly rainfall**			0.140		
Low/medium(<106mm/month)	184 (25.1)	1			
High (>106mm/month)	65 (31.4)	1.25 (0.67-2.31)			
**Sand-soil content (2m)**			0.903		
Low/medium (<555g/1kg)	163 (23.3)	1			
High (>555g/kg)	86 (35.5)	0.95 (0.56-1.61)			

**Coarse fragment content (2m)**			0.022		0.006
Low/medium (<123 cm^3/dm^3)	178 (31.2)	1		1	
High (>123 cm^3/dm^3)	71 (19.1)	0.57 (0.37-0.87)		0.56 (0.37-0.85)	
**Silt-soil content (2m)**			0.391		
Low/medium (<165g/1kg)	151 (32.5)	1			
High (>165g/1kg)	98 (20.6)	1.22 (0.76-1.96)			
**Depth to bedrock (1.75m)**			0.159		
Low/medium (<1.7m)	187 (26.5)	1			
High (>1.7m)	62 (26.3)	0.69 (0.35-1.36)			
**Depth to water table**			0.368		
0-7m	74 (24.5)	1			
7-50m	175 (27.4)	1.12 (0.6-2.1)			
**Enhanced vegetation index**			0.648		
Low/medium (<0.38)	156 (22.6)	1			
High (>0.38)	93 (37.2)	1.22 (0.74-2.01)			
**Slope (incline)**			0.802		
Low/medium (<8%)	156 (25.5)	1			
High (>8%)	92 (28.7)	1.04 (0.72-1.52)			
**Cluster-level sanitation coverage**			0.026		0.017
0-25%	66 (15.6)	1		1	
25-50%	75 (28.6)	1.66 (0.64-4.3)		1.68 (0.69-4.12)	
50-75%	38 (32.2)	1.68 (.55-5.16)		1.93 (0.67-5.31)	
75-100%	70 (50.4)	4.28 (1.42-12.93)		4.77 (1.81-12.61)	
**CLTS triggering**			0.913		
No triggering	240 (27)	1			
Triggered	9 (17.6)	0.71 (0.26-1.9)			
**Access to shared sanitation on other compound**			0.229		
No access	185 (23.5)	1			
Access	64 (41.3)	1.46 (0.93-2.3)			

*Odds ratios and 95% confidence intervals from the full model.

** Global P-values from the full model, outputted from Wald tests.

***Odds ratios and 95% confidence intervals from the final model.

**** Global p-value from final model, outputted from Wald tests.

Table S5. Full and final model outputs measuring associations between households sustaining access to sanitation (sustained adoption) over the study period and contextual, psychosocial and technological factors in 2015

Variable	Proportion with outcome, n (%)	Full model odds ratio (95 CI)*	Full model p-value**	Final model odds ratio (95 CI)***	Final model p-value****
**SES quintile**			0.425		
Lowest wealth	55 (77.5)	1			
2	30 (75)	0.56 (0.2-1.59)			
3	51 (82.3)	1.19 (0.45-3.14)			
4	91 (86.7)	1.14 (0.45-2.87)			
Highest wealth	171 (91.9)	1.55 (0.58-4.16)			
**Head of household sex**			0.505		
Female	93 (82.3)	1			
Male	302 (86.8)	1.25 (0.61-2.55)			
**Education of head of household**			0.319		0.028
No education	85 (77.3)	1		1	
Primary	190 (86.4)	1.63 (0.82-3.23)		1.88 (1-3.47)	
Secondary or above	120 (91.6)	1.9 (0.78-4.64)		2.72 (1.22-6.04)	
**Number of household members**			0.148		
1 to 4	155 (88.1)	1			
5 to 6	134 (86.5)	0.87 (0.42-1.79)			
7+	109 (82)	0.5 (0.24-1.06)			
**Proximity to main road**			0.837		
>4km majroad	39 (86.7)	1			
<4km majroad	359 (85.7)	1.01 (0.35-2.94)			
**Locality type**			0.028		0.002
Rural	286 (88.5)	1		1	
Peri-urban/urban	112 (79.4)	0.37 (0.17-.83)		0.38 (0.21-0.7)	
**Aridity index**			0.565		
semi-arid/sub-sumid	72 (82.8)	1			
Humid	326 (86.5)	0.91 (0.37-2.22)			
**Average monthly rainfall**			0.582		
Low/medium (<106mm/month)	246 (83.7)	1			
High (>106mm/month)	152 (89.4)	1.35 (0.63-2.87)			
**Sand-soil content (2m)**			0.683		
Low/medium (<555g/1kg)	198 (82.2)	1			
High (>555g/kg)	200 (89.7)	0.95 (0.44-2.06)			
**Coarse fragment content (2m)**			0.936		
Low/medium (<123 cm^3/dm^3)	289 (88.1)	1			
High (>123 cm^3/dm^3)	109 (80.1)	0.94 (0.46-1.9)			
**Silt-soil content (2m)**			0.174		

Low/medium (<165g/1kg)	300 (85.7)	1			
High (>165g/1kg)	98 (86)	1.74 (0.78-3.85)			
**Depth to bedrock (1.75m)**			0.879		
low/medium (<1.7m)	235 (83)	1			
High (>1.7m)	160 (89.9)	0.98 (0.44-2.18)			
**Depth to water table**			0.320		
0-7m	130 (81.3)	1			
7-50m	268 (88.2)	1.46 (0.73-2.9)			
**Enhanced vegetation index**			0.706		
Low/medium (<0.38)	185 (80.8)	1			
High (>0.38)	213 (90.6)	1.18 (0.5-2.75)			
**Slope (incline)**			0.766		
Low/medium (<8%)	238 (84.7)	1			
High (>8%)	154 (87)	1.11 (0.59-2.09)			
**Cluster-level sanitation coverage**			0.677		
0-25%	36 (80)	1			
25-50%	68 (77.3)	1.1 (0.37-3.31)			
50-75%	68 (82.9)	1.57 (0.47-5.28)			
75-100%	226 (90.8)	1.93 (0.6-6.22)			
**CLTS triggering**			0.614		
No triggering	373 (86.1)	1			
Triggered	25 (80.6)	1.36 (0.41-4.48)			
**Exclusive access to facility**			0.010		<0.001
shared access on compound	106 (77.4)	1		1	
exclusive access on compound	292 (89.3)	2.39 (1.25-4.56)		2.73 (1.56-4.77)	
**Facility with durable, washable slab**			0.044		0.014
Without slab	184 (82.5)	1		1	
With slab	214 (88.8)	2.09 (0.95-4.61)		2.1 (1.16-3.79)	
**Faeces visible around latrine opening**			0.127		
No faeces present	353 (84.9)	1			
Faeces present	45 (93.8)	2.82 (0.76-10.48)			
**Facility wall**			0.586		
No wall/natural materials	229 (85.1)	1			
Improved materials	169 (86.7)	0.75 (0.32-1.79)			
**Facility roof**			0.784		
No roof/natural materials	202 (84.2)	1			
Improved materials	196 (87.5)	0.9 (0.42-1.92)			

*Odds ratios and 95% confidence intervals from the full model.

** Global P-values from the full model, outputted from Wald tests.

***Odds ratios and 95% confidence intervals from the final model.

**** Global p-value from final model, outputted from Wald tests.

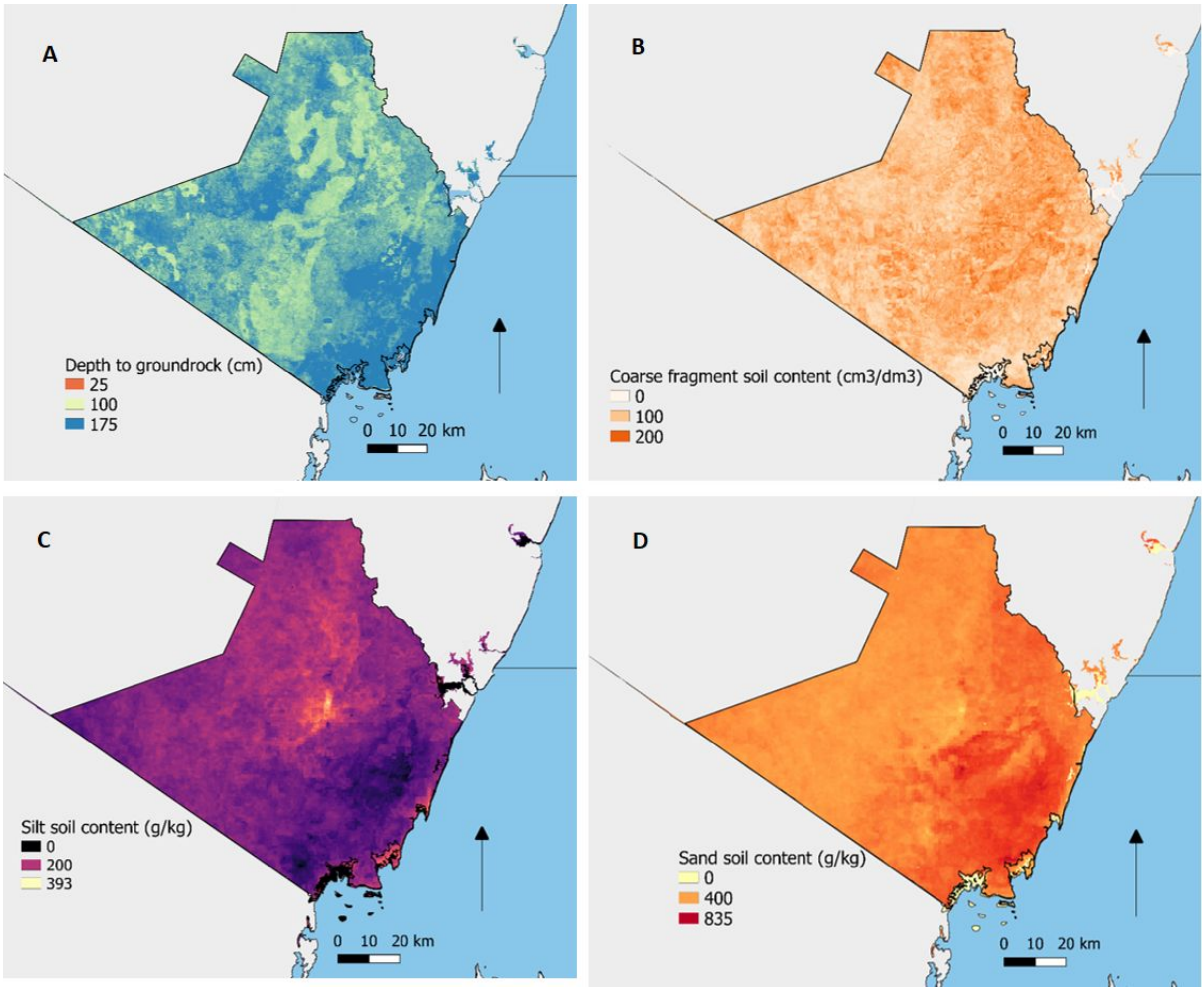

Fig S2. Environmental soil covariates

(A) Depth to groundrock (cm); (B) Coarse fragment soil content (cm3/dm3); (C) Silt soil content (g/kg); (D) Sand soil content (g/kg)

Text S1

Details on environmental covariate data sources and variable creation

Contextual environmental covariates related to soil types included; sand, silt, and coarse fragment content of the soil. Data for sand, silt, and coarse fragment content of the soil were extracted at six depth intervals (0-5cm, 5-15cm, 15-30cm, 30-60cm, 60-100cm, 100-200cm) from the SoilGrids geodatabase (soilgrids.org) and joined with analysis datasets using QGIS (qgis.org). Weighted averages across all depth intervals were calculated before being categorised into tertiles based on the distribution of the data and then dichotomised into high and low/medium. absolute depth to bedrock was also extracted from the SoilGrids database using the same methodology and dichotomised into "shallow" (<170cm) and "deep" (>170cm) depths. Vegetation levels were calculated using the enhanced vegetation index (EVI) measurement and extracted from the Moderate Resolution Imaging Spectroradiometer (MODIS) Instrument [39] before being dichotomised into low/medium levels of vegetation (<0.38) versus high levels of vegetation (>0.38). Slope (percent change in elevation over a given distance) was calculated from a digital elevation matrix extracted from data provided by the Consortium for Spatial Information (CGIAR-CSI) and dichotomised into low/medium (<8%) versus high (>8%). Rainfall data was sourced from the WorldClim (WorldClim.org) database and a 10-year monthly average was calculated before being dichotomised into low/medium (<106mm/month) versus high (>106mm/month). Data on depth to groundwater was accessed from the British Geological Survey and was categorised as shallow (0-7m) versus deep (>7m). Aridity, generated as a function of temperature, rainfall and/or evapotranspiration and dichotomised into "Semi-arid/sub-humid" and "Humid".

Text S2

Details on Factor analysis for generation of relative socioeconomic score

A factor analysis was undertaken to establish a relative socioeconomic score among surveyed households. Separate analyses were conducted with rural and urban households due to differences in the perceived and real value of assets in urban and rural contexts. Following completion, scores were combined and categorised in quintiles. Variables included in both the urban and rural factor analyses included ownership of a motorbike; a bicycle; a mobile phone; a radio; a television; an electricity connection, a sofa; a stone wall for the dwelling, and an improved roof on the dwelling. For both analyses two factors were retained based on the assessment that these factors represented the majority of the variance in the observed variables (Table S6 and Table S8). An oblique rotation was applied given the assumption that factors were correlated (Table S7 and Table S9).

Table S6 – Factor eigenvalues and variance proportions for urban households

Factor	Eigenvalue	Difference	Proportion	Cumulative
Factor1	4.955	4.243	0.903	0.903
Factor2	0.712	0.557	0.130	1.033
Factor3	0.155	0.091	0.028	1.061
Factor4	0.064	0.076	0.012	1.073
Factor5	-0.012	0.017	-0.002	1.071
Factor6	-0.029	0.046	-0.005	1.066
Factor7	-0.075	0.035	-0.014	1.052
Factor8	-0.110	0.066	-0.020	1.032
Factor9	-0.176	.	-0.032	1.000

Table S7 – Rotated factor loadings and unique variances for urban households

Variable	Factor1	Factor2	Uniqueness
Motorbike		0.418	0.693
Bicycle		0.680	0.660
Mobile phone		0.634	0.487
Radio		0.689	0.439
Television	0.723	0.312	0.094
Electricity	0.847		0.081
Sofa	0.750		0.158
Stone wall	0.846		0.360
Metal roof	0.918		0.361

Table S8 – Factor eigenvalues and variance proportions for rural households

Factor	Eigenvalue	Difference	Proportion	Cumulative
Factor1	4.620	3.941	0.920	0.920
Factor2	0.679	0.470	0.135	1.055
Factor3	0.210	0.182	0.042	1.097
Factor4	0.028	0.071	0.006	1.102
Factor5	0.044	0.015	-0.009	1.094
Factor6	0.059	0.044	-0.012	1.082
Factor7	0.103	0.021	-0.021	1.061
Factor8	0.124	0.060	-0.025	1.037
Factor9	0.184	.	-0.037	1.000

Table S9 – Rotated factor loadings and unique variances for rural households

Variable	Factor1	Factor2	Uniqueness
Motorbike		0.413	0.629
Bicycle		0.691	0.633
Mobile phone		0.583	0.574
Radio		0.638	0.451
Television	0.804		0.136
Electricity	0.802		0.226
Sofa	0.825		0.179
Stone wall	0.895		0.336
Metal roof	0.726		0.536

Table S10 – collinearity matrix for covariates included in the sustained adoption model		SES quintile	SES quintile	SES quintile	SES quintile	Head of household sex	Education of head of household	Education of head of household	Number of household members	Number of household members
Variable		2nd	3rd	4th	Highest	male	Primary	Second +	5	7+
SES quintile	2nd	1	0.406682	0.454964	0.45151	-0.07731	-0.06481	0.038043	-0.0822	0.005456
SES quintile	3rd	0.406682	1	0.485745	0.479777	-0.1425	-0.06707	-0.04004	-0.03881	-0.02912
SES quintile	4th	0.454964	0.485745	1	0.587608	-0.20741	-0.03564	-0.08774	0.053793	-0.03271
SES quintile	Highest	0.45151	0.479777	0.587608	1	-0.10101	-0.10284	-0.23215	-0.04021	-0.1108
Head of household sex	male	-0.07731	-0.1425	-0.20741	-0.10101	1	-0.24947	-0.23346	-0.07917	-0.13529
Education of head of household	Primary	-0.06481	-0.06707	-0.03564	-0.10284	-0.24947	1	0.491556	-0.02474	0.050273
Education of head of household	Secondary +	0.038043	-0.04004	-0.08774	-0.23215	-0.23346	0.491556	1	-0.00995	0.043806
Number of household members	5-6	-0.0822	-0.03881	0.053793	-0.04021	-0.07917	-0.02474	-0.00995	1	0.513568
Number of household members	7+	0.005456	-0.02912	-0.03271	-0.1108	-0.13529	0.050273	0.043806	0.513568	1
Proximity to main road	<4km major road	-0.09417	0.031325	0.02665	-0.04671	-0.13925	0.114711	0.036982	0.002115	0.060644
Locality type	Peri-urban/urban	0.158539	-0.03287	0.073308	0.205186	0.06148	-0.01374	0.026258	-0.0004	0.06869
Aridity index	Humid	-0.02301	0.039454	-0.06241	-0.12025	-0.03629	0.003616	0.062101	-0.09103	-0.05252
Average monthly rainfall	High (>106mm/month)	-0.03326	0.008831	-0.06962	0.004219	0.176526	-0.02638	-0.04087	0.025184	0.036776
Sand-soil content (2m)	High (>555g/kg)	0.082324	0.041031	-0.00047	-0.05321	-0.06685	-0.06358	0.059462	0.033085	0.149379
Coarse fragment content (2m)	High (>123cm3/dm3)	-0.05728	-0.01856	0.028371	0.119198	0.077871	-0.06516	-0.06043	0.001431	-0.01754
Silt-soil content (2m)	High (>165g/1kg)	-0.01615	0.064658	-0.03995	-0.05242	-0.01842	0.088417	0.060389	0.00894	0.028202
Depth to bedrock (1.75m)	High (>1.7m)	-0.01692	0.003926	0.048586	-0.02371	-0.10898	-0.04133	-0.03898	0.139054	0.038538
Depth to water table	7-50m	-0.05314	0.062367	0.007314	0.033399	-0.11638	0.014565	0.080183	-0.05431	-0.14993
Enhanced vegetation index	High (>0.38)	-0.07434	-0.1465	-0.08885	-0.04993	0.054243	0.075521	-0.00079	-0.03305	-0.00438
Slope (incline)	High (>8%)	0.064981	0.015409	0.133949	0.116628	0.000705	0.097451	0.008333	0.031491	-0.00451
Cluster-level sanitation	25-50%	-0.00205	-0.00995	0.018889	-0.02287	-0.12134	-0.07622	-0.02169	-0.05275	-0.09008
Cluster-level sanitation	50-75%	-0.04985	-0.01067	0.029189	0.019839	-0.05942	0.024627	-0.01259	0.018747	-0.05319
Cluster-level sanitation	75-100%	-0.0093	0.032465	0.008592	0.021386	0.043545	-0.05916	-0.0922	-0.00491	0.011645
Private access to facility	Private access	-0.09722	-0.04197	-0.10562	-0.09043	-0.01395	0.077702	0.067757	-0.07082	-0.17865
Facility with durable slab	Durable slab	-0.10968	-0.05946	-0.0924	-0.16026	0.006937	0.019359	-0.01258	-0.03592	-0.02796
Faeces around latrine opening	Faeces present	-0.10811	-0.00977	-0.10736	-0.06948	0.077862	-0.07874	-0.0512	-0.02293	-0.08436
Facility wall	Improved	-0.0577	-0.08928	-0.06054	-0.10831	0.085251	-0.04337	-0.01099	-0.00909	-0.03283
Facility roof	Improved	0.008717	-0.01081	-0.04431	-0.13112	-0.02795	0.00498	-0.03868	-0.00768	0.057605

		Proximity to main road	Locality type	Aridity index	Average monthly rainfall	Sand-soil content (2m)	Coarse fragment content	Silt-soil content (2m)	Depth to bedrock (1.75m)	Depth to water table	Enhanced vegetation index
Variable		<4km major road	Peri-urban /urban	Humid	High	High	High	High	High	7-50m	High
SES quintile	2nd	-0.09417	0.158539	-0.02301	-0.03326	0.08232	-0.05728	-0.01615	-0.01692	-0.05314	-0.07434
SES quintile	3rd	0.031325	-0.03287	0.039454	0.008831	0.04103	-0.01856	0.06465	0.00392	0.06236	-0.1465
SES quintile	4th	0.02665	0.073308	-0.06241	-0.06962	-0.00047	0.02837	-0.03995	0.04858	0.00731	-0.08885
SES quintile	Highest	-0.04671	0.205186	-0.12025	0.004219	-0.05321	0.11919	-0.05242	-0.02371	0.03339	-0.04993
Head of household sex	male	-0.13925	0.06148	-0.03629	0.176526	-0.06685	0.07787	-0.01842	-0.10898	-0.11638	0.054243
Education of head of	Primary	0.114711	-0.01374	0.003616	-0.02638	-0.06358	-0.06516	0.08841	-0.04133	0.01456	0.075521
Education of head of	Secondary +	0.036982	0.026258	0.062101	-0.04087	0.05946	-0.06043	0.06038	-0.03898	0.08018	-0.00079
Number of household	5-6	0.002115	-0.0004	-0.09103	0.025184	0.03308	0.00143	0.00894	0.13905	-0.05431	-0.03305
Number of household	7+	0.060644	0.06869	-0.05252	0.036776	0.14937	-0.01754	0.02820	0.03853	-0.14993	-0.00438
Proximity to main road	<4km major road	1	-0.1305	-0.0998	-0.11297	0.04565	0.03418	-0.09777	0.05035	0.08580	0.147048
Locality type	Peri-urban/urban	-0.1305	1	-0.24392	0.015489	0.17915	0.06851	-0.13475	-0.02687	-0.14823	-0.00185
Aridity index	Humid	-0.0998	-0.24392	1	-0.13856	0.07128	-0.02836	0.15544	-0.12473	0.04898	-0.13997
Average monthly rainfall	High	-0.11297	0.015489	-0.13856	1	-0.17164	0.21015	0.15862	-0.09521	0.10692	-0.11211
Sand-soil content (2m)	High (>555g/kg)	0.045658	0.179151	0.071282	-0.17164	1	-0.13305	0.22842	-0.21271	0.00045	-0.20112
Coarse fragment content (2m)	High	0.034184	0.068511	-0.02836	0.210154	-0.13305	1	-0.02337	0.09201	0.10198	0.06523
Silt-soil content (2m)	High (>165g/1kg)	-0.09777	-0.13475	0.155444	0.158627	0.22842	-0.02337	1	-0.18691	0.05409	-0.00617
Depth to bedrock (1.75m)	High (>1.7m)	0.050353	-0.02687	-0.12473	-0.09521	-0.21271	0.09201	-0.18691	1	-0.00286	-0.28599
Depth to water table	7-50m	0.085801	-0.14823	0.048986	0.106929	0.00045	0.10198	0.05409	-0.00286	1	-0.13451
Enhanced vegetation index	High (>0.38)	0.147048	-0.00185	-0.13997	-0.11211	-0.20112	0.06523	-0.00617	-0.28599	-0.13451	1
Slope (incline)	High (>8%)	0.018599	0.185713	-0.11635	-0.05058	-0.00615	-0.03619	0.00575	0.11050	0.00551	0.01089
Cluster-level sanitation	25-50%	-0.13921	-0.33664	-0.06776	0.013741	-0.09029	0.00930	0.02814	0.04375	0.17496	-0.0716
Cluster-level sanitation	50-75%	-0.16249	-0.28771	-0.19099	-0.03651	-0.21165	0.06920	0.1346	0.01373	-0.08683	0.065822
Cluster-level sanitation	75-100%	-0.17733	-0.14627	-0.24364	0.13945	-0.15546	0.15976	0.09457	-0.02301	-0.09672	-0.22929
Private access to facility	Private access	-0.00463	-0.10607	0.041681	-0.1881	-0.02121	0.13549	0.06254	-0.01999	-0.09455	-0.00632
Facility with durable slab	Durable slab	0.039215	-0.25194	0.017037	-0.00519	-0.05166	0.06699	-0.09431	-0.02877	0.02151	0.040422
Faeces around latrine opening	Faeces present	-0.03106	0.030932	0.047696	0.057786	0.00965	-0.02393	0.04755	0.01984	-0.03099	-0.15327
Facility wall	Improved	-0.02971	-0.09939	0.065665	-0.10125	0.07965	-0.05253	-0.08104	-0.09294	-0.03338	0.008421
Facility roof	Improved	0.052606	-0.00772	0.100907	0.0425	-0.02016	-0.1193	0.03931	0.07427	0.00190	-0.0219

		Slope (incline)	Cluster-level sanitation coverage	Cluster-level sanitatio n	Cluster-level sanitatio n	Private access to facility	Facility with durable, washabl	Faeces visible around latrine	Facility wall	Facility roof
Variable		High	25-50%	50-75%	75-100%	Private access	Durable slab	Faeces present	Improved	Improved
SES quintile	2nd	0.064981	-0.00205	-0.04985	-0.0093	-0.09722	-0.10968	-0.10811	-0.0577	0.00871
SES quintile	3rd	0.015409	-0.00995	-0.01067	0.032465	-0.04197	-0.05946	-0.00977	-0.08928	-0.01081
SES quintile	4th	0.133949	0.018889	0.029189	0.008592	-0.10562	-0.0924	-0.10736	-0.06054	-0.04431
SES quintile	Highest	0.116628	-0.02287	0.019839	0.021386	-0.09043	-0.16026	-0.06948	-0.10831	-0.13112
Head of household sex	male	0.000705	-0.12134	-0.05942	0.043545	-0.01395	0.00693	0.07786	0.08525	-0.02795
Education of head of	Primary	0.097451	-0.07622	0.024627	-0.05916	0.07770	0.01935	-0.07874	-0.04337	0.00498
Education of head of	Secondary +	0.008333	-0.02169	-0.01259	-0.0922	0.06775	-0.01258	-0.0512	-0.01099	-0.03868
Number of household	5-6	0.031491	-0.05275	0.018747	-0.00491	-0.07082	-0.03592	-0.02293	-0.00909	-0.00768
Number of household	7+	-0.00451	-0.09008	-0.05319	0.011645	-0.17865	-0.02796	-0.08436	-0.03283	0.05760
Proximity to main road	<4km major road	0.018599	-0.13921	-0.16249	-0.17733	-0.00463	0.03921	-0.03106	-0.02971	0.05260
Locality type	Peri-urban/urban	0.185713	-0.33664	-0.28771	-0.14627	-0.10607	-0.25194	0.03093	-0.09939	-0.00772
Aridity index	Humid	-0.11635	-0.06776	-0.19099	-0.24364	0.04168	0.01703	0.04769	0.06566	0.10090
Average monthly rainfall	High	-0.05058	0.013741	-0.03651	0.13945	-0.1881	-0.00519	0.05778	-0.10125	0.0425
Sand-soil content (2m)	High (>555g/kg)	-0.00615	-0.09029	-0.21165	-0.15546	-0.02121	-0.05166	0.00965	0.07965	-0.02016
Coarse fragment content (2m)	High	-0.03619	0.009308	0.069205	0.159762	0.13549	0.06699	-0.02393	-0.05253	-0.1193
Silt-soil content (2m)	High (>165g/1kg)	0.005755	0.028145	0.1346	0.094572	0.06254	-0.09431	0.04755	-0.08104	0.03931
Depth to bedrock (1.75m)	High (>1.7m)	0.110503	0.04375	0.013735	-0.02301	-0.01999	-0.02877	0.01984	-0.09294	0.07427
Depth to water table	7-50m	0.005512	0.17496	-0.08683	-0.09672	-0.09455	0.02151	-0.03099	-0.03338	0.00190
Enhanced vegetation index	High (>0.38)	0.01089	-0.0716	0.065822	-0.22929	-0.00632	0.04042	-0.15327	0.00842	-0.0219
Slope (incline)	High (>8%)	1	-0.11171	-0.06546	-0.09385	-0.01411	-0.07302	-0.01662	0.09541	-0.06591
Cluster-level sanitation	25-50%	-0.11171	1	0.654821	0.651855	-0.07406	0.1468	-0.02605	0.02649	-0.03104
Cluster-level sanitation	50-75%	-0.06546	0.654821	1	0.693315	0.02096	0.04741	-0.04788	0.01281	-0.01451
Cluster-level sanitation	75-100%	-0.09385	0.651855	0.693315	1	-0.06965	0.07796	0.01746	0.00655	-0.0466
Private access to facility	Private access	-0.01411	-0.07406	0.020966	-0.06965	1	0.05588	0.10378	0.03322	-0.07097
Facility with durable slab	Durable slab	-0.07302	0.1468	0.047412	0.077964	0.05588	1	0.06417	-0.38279	-0.15439
Faeces around latrine opening	Faeces present	-0.01662	-0.02605	-0.04788	0.017468	0.10378	0.06417	1	-0.04454	0.05376
Facility wall	Improved	0.095419	0.026492	0.012815	0.006551	0.03322	-0.38279	-0.04454	1	-0.42169
Facility roof	Improved	-0.06591	-0.03104	-0.01451	-0.0466	-0.07097	-0.15439	0.05376	-0.42169	1

Table S11 – collinearity matrix for covariates included in the initial adoption model		SES quintile	SES quintile	SES quintile	SES quintile	Head of household sex	Education of head of household	Education of head of household	Number of household members	Number of household members	Proximity to main road
Variable		2nd	3rd	4th	Highest	male	Primary	Secondary +	05-Jun	7+	<4km major road
SES quintile	2nd	1	0.359629	0.349953	0.305248	-0.06073	0.001164	-0.04473	-0.0246	-0.05292	0.015364
SES quintile	3rd	0.359629	1	0.404759	0.349635	-0.05397	-0.1186	-0.08365	-0.07353	-0.07416	-0.00434
SES quintile	4th	0.349953	0.404759	1	0.358411	-0.08893	-0.08672	-0.16843	-0.06165	-0.07621	0.024376
SES quintile	Highest	0.305248	0.349635	0.358411	1	-0.03404	-0.10706	-0.22737	-0.04281	-0.05378	-0.04851
Head of household sex	male	-0.06073	-0.05397	-0.08893	-0.03404	1	-0.35614	-0.23222	-0.04614	-0.12215	-0.02249
Education head household	Primary	0.001164	-0.1186	-0.08672	-0.10706	-0.35614	1	0.439476	-0.02073	-0.04203	0.038924
Education head household	Secondary +	-0.04473	-0.08365	-0.16843	-0.22737	-0.23222	0.439476	1	-0.02191	0.040893	-0.01098
Nbr of household members	5-6	-0.0246	-0.07353	-0.06165	-0.04281	-0.04614	-0.02073	-0.02191	1	0.472138	-0.00643
Nbr of household members	7+	-0.05292	-0.07416	-0.07621	-0.05378	-0.12215	-0.04203	0.040893	0.472138	1	0.019159
Proximity to main road	<4km major road	0.015364	-0.00434	0.024376	-0.04851	-0.02249	0.038924	-0.01098	-0.00643	0.019159	1
Locality type	Peri-urban/urban	0.089329	0.110271	0.0874	0.139735	0.011364	-0.01443	-0.06821	-0.04242	-0.03878	-0.04791
Aridity index	Humid	-0.00162	-0.00745	-0.02155	-0.00864	-0.02019	-0.06096	-0.04723	0.018495	0.045966	-0.03562
Average monthly rainfall	High	0.0194	0.008581	-0.02451	0.006404	0.032308	0.023832	-0.0061	0.044161	0.031788	-0.10892
Sand-soil content (2m)	High (>555g/kg)	-0.02847	-0.02238	0.039514	-0.01351	0.007061	-0.11046	-0.09057	0.029221	0.04777	0.066677
Coarse fragment content	High	-0.04608	0.001866	-0.01467	0.008724	-0.0469	0.008129	0.013045	0.028795	-0.03509	-0.06169
Silt-soil content (2m)	High (>165g/1kg)	-0.04679	-0.03499	-0.01849	-0.03396	-0.03313	-0.01607	-0.01164	0.035841	0.082121	0.01717
Depth to bedrock (1.75m)	High (>1.7m)	-0.01329	0.018815	0.026778	0.050901	-0.01629	0.053018	-0.00504	-0.03587	-0.03299	-0.13738
Depth to water table	7-50m	-0.02063	0.022368	0.021666	-0.00856	-0.01731	-0.02055	0.03864	0.003885	-0.00967	0.134897
Enhanced vegetation index	High (>0.38)	-0.03981	-0.04764	-0.09087	-0.00677	-0.01117	0.009095	0.055779	0.003823	0.016552	0.035768
Slope (incline)	High (>8%)	-0.03325	-0.02995	-0.03368	-0.0085	0.063197	-0.03936	-0.02603	0.036557	0.000692	-0.00154
Cluster-level sanitation	25-50%	-0.02656	0.005457	-0.01483	0.01652	-0.01152	-0.0196	0.017123	0.004417	0.009491	-0.22836
Cluster-level sanitation	50-75%	-0.04609	-0.03883	-0.00545	-0.02089	0.026085	0.002112	0.031438	0.011513	-0.00709	-0.21684
Cluster-level sanitation	75-100%	-0.02994	-0.01668	0.015361	0.019523	0.033082	0.01886	0.003069	-0.00388	0.00663	-0.18505
Shared access off compound	With access	-0.04089	-0.00158	-0.0238	0.025309	0.065751	-0.06988	-0.05842	-0.00406	0.011367	-0.06705

		Locality type	Aridity index	Average monthly rainfall	Sand-soil content (2m)	Coarse fragment content (2m)	Silt-soil content (2m)	Depth to bedrock (1.75m)	Depth to water table	Enhanced vegetation index	Slope (incline)
Variable		Peri-urban/ urban	Humid	High	High	High	High	High	7-50m	High	High
SES quintile	2nd	0.089329	-0.00162	0.0194	-0.02847	-0.04608	-0.04679	-0.01329	-0.02063	-0.03981	-0.03325
SES quintile	3rd	0.110271	-0.00745	0.008581	-0.02238	0.001866	-0.03499	0.018815	0.022368	-0.04764	-0.02995
SES quintile	4th	0.0874	-0.02155	-0.02451	0.039514	-0.01467	-0.01849	0.026778	0.021666	-0.09087	-0.03368
SES quintile	Highest	0.139735	-0.00864	0.006404	-0.01351	0.008724	-0.03396	0.050901	-0.00856	-0.00677	-0.0085
Head of household sex	male	0.011364	-0.02019	0.032308	0.007061	-0.0469	-0.03313	-0.01629	-0.01731	-0.01117	0.063197
Education head household	Primary	-0.01443	-0.06096	0.023832	-0.11046	0.008129	-0.01607	0.053018	-0.02055	0.009095	-0.03936
Education head household	Secondary +	-0.06821	-0.04723	-0.0061	-0.09057	0.013045	-0.01164	-0.00504	0.03864	0.055779	-0.02603
Nbr of household members	5-6	-0.04242	0.018495	0.044161	0.029221	0.028795	0.035841	-0.03587	0.003885	0.003823	0.036557
Nbr of household members	7+	-0.03878	0.045966	0.031788	0.04777	-0.03509	0.082121	-0.03299	-0.00967	0.016552	0.000692
Proximity to main road	<4km major road	-0.04791	-0.03562	-0.10892	0.066677	-0.06169	0.01717	-0.13738	0.134897	0.035768	-0.00154
Locality type	Peri-urban/urban	1	-0.22347	-0.0534	0.085829	0.146128	-0.11045	0.068273	-0.05272	-0.0193	0.039155
Aridity index	Humid	-0.22347	1	-0.19692	0.013816	-0.01669	0.159187	-0.14452	0.092584	-0.06003	0.067488
Average monthly rainfall	High	-0.0534	-0.19692	1	-0.21109	0.091568	0.03875	-0.07079	0.061873	0.011893	0.050069
Sand-soil content (2m)	High (>555g/kg)	0.085829	0.013816	-0.21109	1	-0.00602	0.249522	-0.20254	0.026442	-0.06914	0.045917
Coarse fragment content	High	0.146128	-0.01669	0.091568	-0.00602	1	-0.11475	0.045304	0.027955	0.046286	-0.00603
Silt-soil content (2m)	High (>165g/1kg)	-0.11045	0.159187	0.03875	0.249522	-0.11475	1	-0.01696	-0.01881	0.012726	0.00597
Depth to bedrock (1.75m)	High (>1.7m)	0.068273	-0.14452	-0.07079	-0.20254	0.045304	-0.01696	1	-0.07393	-0.23877	-0.03668
Depth to water table	7-50m	-0.05272	0.092584	0.061873	0.026442	0.027955	-0.01881	-0.07393	1	-0.07866	0.045092
Enhanced vegetation index	High (>0.38)	-0.0193	-0.06003	0.011893	-0.06914	0.046286	0.012726	-0.23877	-0.07866	1	0.030738
Slope (incline)	High (>8%)	0.039155	0.067488	0.050069	0.045917	-0.00603	0.00597	-0.03668	0.045092	0.030738	1
Cluster-level sanitation	25-50%	-0.16595	-0.11013	0.036877	-0.02299	0.01565	0.081632	0.03537	0.081189	-0.06268	-0.06515
Cluster-level sanitation	50-75%	-0.18022	-0.12947	-0.07622	-0.05831	0.022246	0.124712	-0.00154	-0.0988	-0.03857	-0.03559
Cluster-level sanitation	75-100%	-0.07019	-0.26891	0.038549	-0.06309	0.037432	0.117376	0.044651	-0.08675	-0.18058	-0.09864
Shared access off compound	With access	-0.08849	-0.00369	0.083778	-0.08679	-0.05568	0.012096	-0.0032	-0.03902	0.010839	0.086497

Variable		Cluster-level sanitation coverage 25-50%	Cluster-level sanitation coverage 50-75%	Cluster-level sanitation coverage 75-100%	Shared access off compound With access
SES quintile	2nd	-0.02656	-0.04609	-0.02994	-0.04089
SES quintile	3rd	0.005457	-0.03883	-0.01668	-0.00158
SES quintile	4th	-0.01483	-0.00545	0.015361	-0.0238
SES quintile	Highest	0.01652	-0.02089	0.019523	0.025309
Head of household sex	male	-0.01152	0.026085	0.033082	0.065751
Education of head of	Primary	-0.0196	0.002112	0.01886	-0.06988
Education of head of	Secondary +	0.017123	0.031438	0.003069	-0.05842
Number of household	5-6	0.004417	0.011513	-0.00388	-0.00406
Number of household	7+	0.009491	-0.00709	0.00663	0.011367
Proximity to main road	<4km major road	-0.22836	-0.21684	-0.18505	-0.06705
Locality type	Peri-urban/urban	-0.16595	-0.18022	-0.07019	-0.08849
Aridity index	Humid	-0.11013	-0.12947	-0.26891	-0.00369
Average monthly rainfall	High	0.036877	-0.07622	0.038549	0.083778
Sand-soil content (2m)	High (>555g/kg)	-0.02299	-0.05831	-0.06309	-0.08679
Coarse fragment content	High	0.01565	0.022246	0.037432	-0.05568
Silt-soil content (2m)	High (>165g/1kg)	0.081632	0.124712	0.117376	0.012096
Depth to bedrock (1.75m)	High (>1.7m)	0.03537	-0.00154	0.044651	-0.0032
Depth to water table	7-50m	0.081189	-0.0988	-0.08675	-0.03902
Enhanced vegetation	High (>0.38)	-0.06268	-0.03857	-0.18058	0.010839
Slope (incline)	High (>8%)	-0.06515	-0.03559	-0.09864	0.086497
Cluster-level sanitation	25-50%	1	0.510758	0.521838	-0.00411
Cluster-level sanitation	50-75%	0.510758	1	0.519081	-0.07981
Cluster-level sanitation	75-100%	0.521838	0.519081	1	-0.06679
Private access to facility	Private access	-0.00411	-0.07981	-0.06679	1
